# A distinct LHCI arrangement is recruited to photosystem I in Fe-starved green algae

**DOI:** 10.1073/pnas.2500621122

**Published:** 2025-06-16

**Authors:** Helen W. Liu, Radhika Khera, Patricia Grob, Sean D. Gallaher, Samuel O. Purvine, Carrie D. Nicora, Mary S. Lipton, Krishna K. Niyogi, Eva Nogales, Masakazu Iwai, Sabeeha S. Merchant

**Affiliations:** ^a^Department of Plant and Microbial Biology, University of California, Berkeley, CA 94720; ^b^HHMI, University of California, Berkeley, CA 94720; ^c^California Institute for Quantitative Biosciences, University of California, Berkeley, CA 94720; ^d^Department of Molecular and Cell Biology, University of California, Berkeley, CA 94720; ^e^Earth and Biological Sciences Division, Pacific Northwest National Laboratory, Richland, WA 99352; ^f^Molecular Biophysics and Integrated Bioimaging Division, Lawrence Berkeley National Laboratory, Berkeley, CA 94720; ^g^Environmental Genomics and Systems Biology, Lawrence Berkeley National Laboratory, Berkeley, CA 94720

**Keywords:** iron homeostasis, TIDI, phytoplankton, structural biology, iron starvation–induced protein A (isiA)

## Abstract

Iron (Fe) availability limits photosynthesis at a global scale where Fe-rich photosystem (PS) I abundance is drastically reduced in Fe-poor environments. We used single-particle cryoelectron microscopy to reveal a unique Fe starvation-dependent arrangement of light-harvesting chlorophyll (LHC) proteins where Fe starvation–induced TIDI1 is found in an additional tetramer of LHC proteins associated with PSI in *Dunaliella tertiolecta* and *Dunaliella salina*. These cosmopolitan green algae are resilient to poor Fe nutrition. TIDI1 is a distinct LHC protein that co-occurs in diverse algae with flavodoxin (an Fe-independent replacement for the Fe-containing ferredoxin). The antenna expansion in eukaryotic algae we describe here is reminiscent of the iron-starvation induced (*isiA-*encoding) antenna ring in cyanobacteria, which typically co-occurs with *isiB*, encoding flavodoxin. Our work showcases the convergent strategies that evolved after the Great Oxidation Event to maintain PSI capacity.

## Significance

Photosynthetic proteins require a significant amount of iron (Fe)-containing cofactors to function. Multisubunit Photosystem I (PSI) encompasses half of this Fe in its three Fe_4_S_4_ centers and is a target for degradation in insufficient Fe conditions, which reduces the capacity for CO_2_ fixation. TIDI1 (thylakoid iron deficiency induced) is an iron starvation induced chlorophyll-binding protein found in multiple green algae including *Dunaliella* spp. isolated from coastal Arctic waters and hypersaline ponds. These algae are especially resilient to low Fe conditions. Using single particle cryo-EM, we show PSI from Fe-starved *Dunaliella* spp. has a second light-harvesting chlorophyll protein tetramer containing TIDI1, showcasing a eukaryotic strategy to maintain PSI efficiency in low iron.

About half of Earth’s primary productivity occurs through photosynthesis by marine algae (1). The photosynthetic machinery evolved in an Fe-rich anoxic world, but in today’s oxygenated world, Fe-bioavailability is poor due to the low solubility of ferric species (2, 3). Three multisubunit membrane protein complexes in photosynthesis, photosystem (PS) II, the cytochrome (Cyt) *b*_6_*f* complex, and PSI, catalyze sequential electron transfer from water to generate reductant for biosynthesis, and all require Fe as a cofactor. These complexes are a major Fe sink in the cell given their intracellular abundance and their high Fe stoichiometry (4–6). PSI has the highest stoichiometry, with three iron-sulfur (Fe_4_S_4_) clusters per monomer, corresponding to about half the Fe in the photosynthetic apparatus, and it is thus disproportionately impacted by poor Fe nutrition. Consequently, the PSI content of green algae, diatoms, and cyanobacteria is reduced substantially under Fe starvation, with an impact on photosynthetic productivity and biomass. But little else is known about the molecular mechanisms of algal PSI adaptations to low Fe conditions in the ocean.

**Fig. 1.**
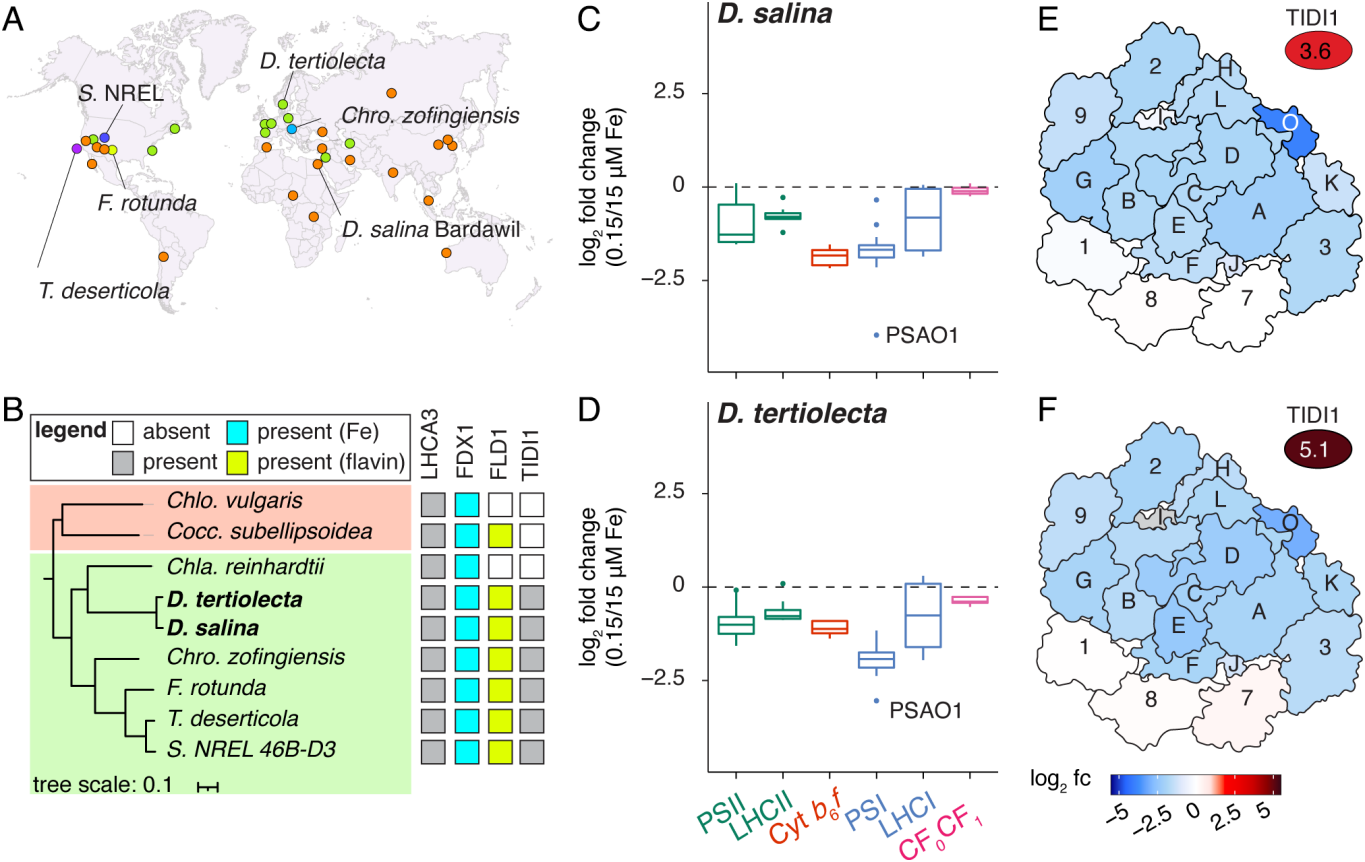
Uneven response of *Dunaliella* spp. LHCA subunits in Fe-starved medium. (*A*) Geographic distribution of TIDI-encoding algae of either *D. salina* (orange), *D. tertiolecta* (green), *Chro. zofingiensis* (dark blue), *T. deserticola* (purple), *F. rotunda* (yellow), and *S. NREL 46B-D3* (light blue) modified from Davidi et al. (8). The strains used in this work are *D. tertiolecta* UTEX LB999 and *D. salina* Bardawil UTEX LB 2538. (*B*) A species tree showing the relationship between TIDI1 and non-TIDI1 encoding species from Chlorophyceae (green) and Trebouxiophyceae (salmon). The tree is based on the concatenated polypeptide sequences of universal single-copy orthologs (USCOs) shared among all 9 species. Filled squares signifies the presence of at least one homolog of the indicated protein (FDX1, ferredoxin; FLD1, flavodoxin; LHCA3; TIDI1). Square color describes proteins that are either present (gray), present and binds Fe (cyan), present and binds flavin (green), or absent (white). (*C* and *D*) The log_2_ fold changes (fc) of protein abundances in the Fe-replete (15 µM Fe) and Fe-starved (0.15 µM Fe) condition detected by at least two spectral counts encoding components of PSII, LHCII, Cyt *b*_6_*f*, PSI, LHCI, and CF_O_CF_1_ (ATP synthase) for *D. salina* (*C*) and *D. tertiolecta* (*D*). For a complete list of protein subunits and their abundances, see Dataset S2. (*E* and *F*) The PSI (PSA) and LHCI (LHCA) subunits of PDB:6SL5 (stromal view) colored by its log_2_ fold changes (fc) of protein abundances (red, increase; blue, decrease) for *D. salina* (*E*) and *D. tertiolecta* (*F*). Gray values indicate not detected (<2 spectral counts).

To address this gap, we surveyed the globally abundant, cosmopolitan green algae, *Dunaliella* spp. (Fig. 1*A*). These algae are obligate photoautotrophs, that tolerate extreme pH, salinity, light, and temperature, making them compelling organisms to investigate the diversity of both photoacclimation and Fe starvation response mechanisms (7). *Dunaliella* spp. are well adapted to low Fe conditions, maintaining photosynthesis at Fe concentrations that are limiting to other photosynthetic organisms (*SI Appendix*, Fig. S1). Here, we used two halotolerant *Dunaliella* spp. that diverged ~253 million years ago: *D. tertiolecta* (isolated from the coastal Arctic water environment of Oslofjord, Norway) and *D. salina* (isolated from hypersaline Lake Bardawil in Egypt) (Fig. 1*A*) (8). Both organisms have an expanded repertoire of Fe homeostasis genes and express a range of similar Fe starvation responses that enables productivity under Fe-poor conditions, including upregulation of Fe-acquisition pathways, replacement of Fe-containing ferredoxin with flavin-containing flavodoxin, and downregulation of the Fe-containing photosynthetic apparatus, especially PSI components (8–13).

In contrast to the decreased expression of PSI subunits, the expression of one thylakoid membrane protein, named TIDI1 (thylakoid iron deficiency induced), is dramatically increased under Fe starvation, similarly to the expression of FLD1, a flavodoxin (*SI Appendix*, Fig. S1 *G* and *H*) (8, 14). A survey of green algal genomes found TIDI1 orthologs in a subset of green algae: *Chromochloris zofingiensis*, *Flechtneria rotunda*, *Tetradesmus deserticola*, and *Scenedesmus* sp. NREL 46B-D3 (8) (Fig. 1*B*). These algae were isolated from diverse habitats, including fresh water, brackish waters, and the desert, indicating that TIDI1 is neither a species-specific nor an environment-specific adaptation (7, 15, 16) (Fig. 1 *A* and *B*). As an intriguing correlation, each of these organisms also contains an ortholog of eukaryotic FLD1 (Fig. 1*B*), consistent with the cofunction of TIDI1 and FLD1 in a low Fe environment (8, 17). Sequence analysis places TIDI1 in the family of light-harvesting chlorophyll (LHC) proteins (8, 14, 18). TIDI1 shares ~50% sequence similarity to LHCA3s, which occur universally in the green lineage (8, 14, 18). Among its unique features, TIDI1 has a proline-rich stromal N-terminal domain and a longer lumenal loop between the transmembrane helices B and C (8, 14). TIDI1 comigrates with PSI-LHCI supercomplexes from Fe-starved *D. tertiolecta*, suggesting that TIDI1 may function in an Fe starvation-modified antenna system (14). 77 K fluorescence emission spectra of these PSI-LHCI supercomplexes from Fe-starved *D. tertiolecta* indicated a larger antenna that is functionally coupled to the PSI reaction center (14). However, the nature of the association and the placement of TIDI1 within the green algal antenna are not known.

In this work, we report high-resolution cryoelectron microscopy (cryo-EM) structures of PSI-LHCI supercomplexes from Fe-starved compared to Fe-replete cells of both *D. salina* and *D. tertiolecta*. We discovered that the PSI-LHCI supercomplexes under Fe-starved conditions have an additional LHCI tetramer in which TIDI1 replaces LHCA3 in the new tetramer. These structures represent further examples of LHC arrangements and illustrate the diversity of PSI-LHCI supercomplexes among photosynthetic organisms. This work constitutes a structural depiction of eukaryotic PSI-LHCI antenna remodeling, which has not been previously described as an adaptive mechanism in response to Fe starvation.

## Results

### LHCA1, LHCA7, and LHCA8 are Selectively Maintained in Fe-Starved Medium.

To quantify the changes in abundance of the proteins of the photosynthetic machinery under Fe starvation, we performed tandem mass tag (TMT) quantitative proteomics using cultures grown in Fe-replete (15 µM Fe) and Fe-starved (0.15 µM Fe) conditions. The most dramatic changes are for PSI, whose subunits decrease about 4-fold relative to the Fe-replete *Dunaliella* cells (Fig. 1 *C* and *D* and *SI Appendix*, Fig. S2). This result is not unexpected given PSI’s high Fe content. By contrast, PSII, which requires only two Fe atoms per core monomer, showed less decrease (Fig. 1 *C* and *D*). PSI and PSII are each associated with LHC proteins for increased absorption cross-section, LHCA for PSI and LHCB (or LHCBM) for PSII. These proteins are organized in the LHCI and LHCII oligomeric complexes, respectively. When we monitored the abundances of LHCI and LHCII subunits, we noted that the six *Dunaliella* LHCA proteins (numbered according to *Chlamydomonas reinhardtii* LHCA orthologs) are, like the PSA subunits, more substantially reduced in low Fe relative to the PSII (PSB) subunits and LHCB proteins (Fig. 1 *C* and *D*). Nevertheless, quantitative proteomics data indicated a striking difference between the abundance of three LHCA proteins (LHCA1, LHCA8, and LHCA7), which remain unchanged, and the other LHCA proteins (LHCA2, LHCA3, and LHCA9), which decreased proportionately with PSI subunits, as expected (Fig. 1 *E* and *F*). TIDI1, on the other hand, was greatly increased in low Fe medium in both *Dunaliella* spp., as previously shown (8, 14). The changes in protein abundance mirror changes in transcript abundances (8), indicative of an antenna remodeling program in Fe starvation (Fig. 1 *E* and *F*). These results motivated structural analysis of PSI-LHCI supercomplexes.

### Isolation of Dunaliella PSI-LHCI Supercomplexes from Fe-Replete and Fe-Starved Cells.

We isolated PSI-LHCI supercomplexes from Fe-replete (15 µM Fe) and Fe-starved (0.15 µM Fe) cultures of both *Dunaliella* spp. (see Supplemental Methods and *SI Appendix*, Fig. S3 *A* and *D*) using density gradient centrifugation of detergent-solubilized thylakoid membranes. The PSI supercomplexes from Fe-starved cells appeared in denser fractions of the gradient compared to those from Fe-replete cells (*SI Appendix*, Fig. S3 *A* and *D*), with *D. salina* separating in even denser fractions relative to *D. tertiolecta*, intimating larger PSI-LHCI supercomplexes in the Fe-starved situations. Immunoblot analysis indicated the presence of TIDI1 in these denser fractions (*SI Appendix*, Fig. S3 *B* and *E*). Low-temperature fluorescence emission spectra of each PSI-containing fraction exhibited a single peak around 710 nm, confirming the purity of the isolated PSI-LHCI supercomplexes, with the peak from the Fe-starved cultures being 2 to 3 nm blue-shifted (*SI Appendix*, Fig. S3 *C* and *F*), perhaps reflecting a change in PSI antenna organization as hypothesized previously for PSI-LHCI supercomplexes from Fe-starved *D. tertiolecta* cells (14, 19).

### Overall Structures of Dunaliella spp. PSI-LHCI Supercomplexes from Fe-Replete Cells.

**Fig. 2.**
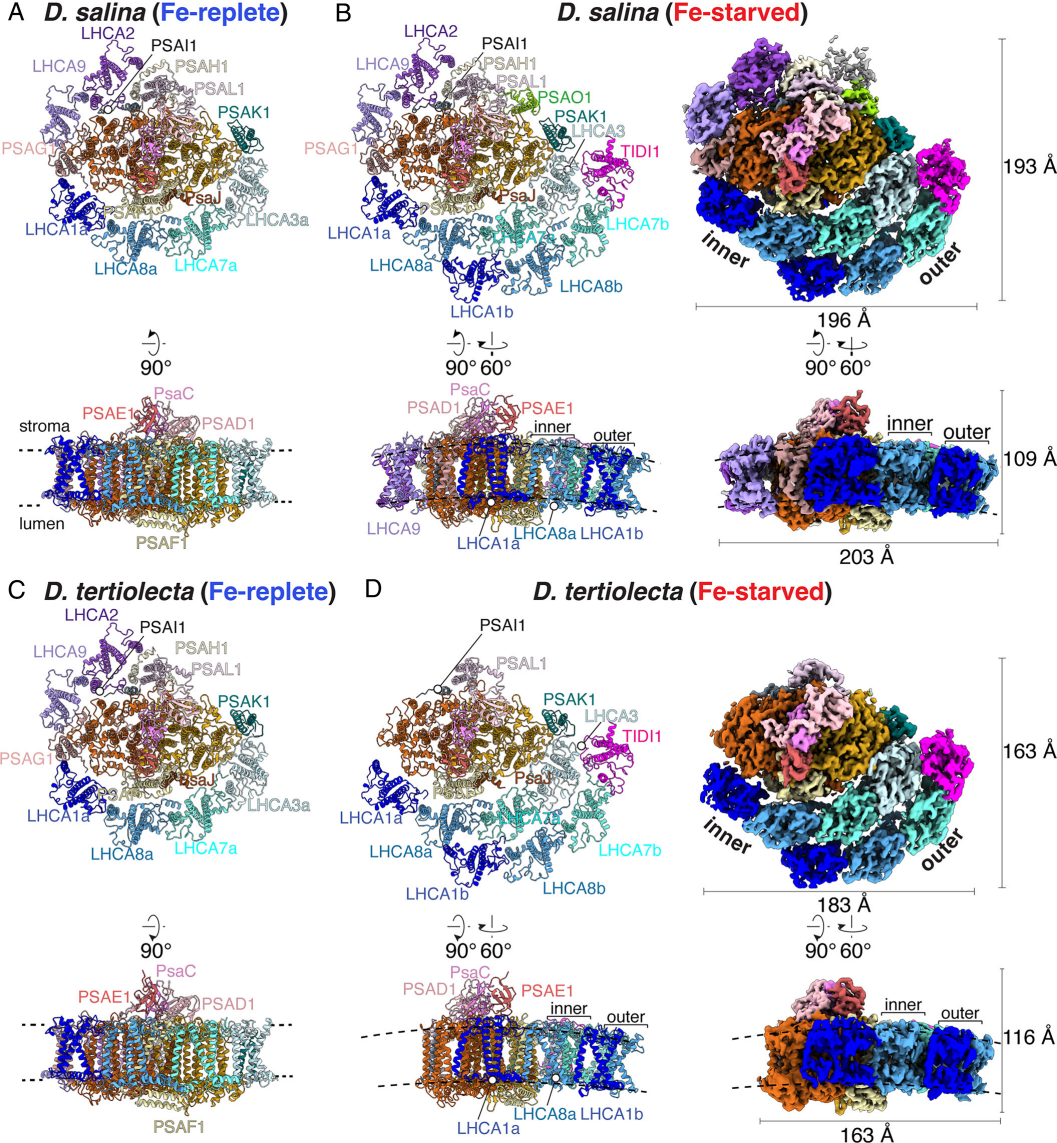
Comparison of the Fe-replete and Fe-starved *Dunaliella* PSI-LHCI supercomplexes. Stromal view (*Top*) or side view (*Bottom*) of the PSI-LHCI supercomplexes from *D. salina* (*A* and *B*) and *D. tertiolecta* (*C* and *D*). (*A*) Fe-replete *Ds*PSI-LHCI_1_ supercomplex and (*C*) Fe-replete *Dt*PSI–LHCI_1_ supercomplex in ribbon mode. (*B* and *D*) Fe-starved *Ds*PSI–LHCI_2_ supercomplex (B) and *Dt*PSI–LHCI_2_ supercomplex (*D*) in ribbon mode (*Left*) and the composite cryo-EM density map (*Right*). Subunits are shown in different colors and labeled. Membrane-extrinsic subunits PsaC, PSAD, and PSAE are labeled only in the side view. Black dashed lines indicated the membrane-spanning region of the PSI-LHCI supercomplexes.

We used single-particle cryo-EM to determine the structures of both *D. salina* (*Ds*) and *D. tertiolecta* (*Dt*) PSI-LHCI supercomplexes from Fe-replete cultures as exemplars of the native state (hereafter named PSI-LHCI_1_) at overall resolutions of 2.9 Å and 2.1 Å, respectively (See Methods and Fig. 2 *A* and *C* and *SI Appendix*, Figs. S4, S5, and S6 and Table S1). The density maps were better defined around the PSI core than near the detergent belt (*SI Appendix*, Figs. S4, S5, and S6), consistent with a higher level of flexibility in the outer regions of the complex. Three subcomplex features are evident in the structures: the PSI core, consisting of all but one of the 12 PSA subunits encoded in the genome (PsaA to PSAL1); one crescent-shaped LHCI tetramer made up of four LHCA subunits (LHCA1, LHCA8, LHCA7, and LHCA3) arranged from the PSAG1 (proximal) to the PSAK1 (distal) side; and one LHCI dimer (LHCA2 and LHCA9) on the opposite side, together accounting for all 6 LHCA proteins encoded in the genome (*SI Appendix*, Table S2). We lacked density for the PSAO1 subunit, which may be attributed to our isolation or vitrification conditions [Fig. 2*A* and *SI Appendix*, Fig. S6, (20)], since the protein is detected by proteomics (Fig. 1 *E* and *F*) albeit with undetermined occupancy in the PSI-LHCI supercomplex. Among the ~300,000 particles evaluated, we did not find a previously reported *D. salina* “minimal” PSI-LHCI supercomplex lacking the LHCI dimer, PSAG1, PSAH1, PSAI1, PSAL1, and PSAO1 (*SI Appendix*, Figs. S4 and S5) (21). Otherwise, our structure recapitulates the more typical PSI supercomplex structure (20). The coordinate decrease of all PSA subunits in Fe-starved cells, evident in whole-cell proteomics data (Fig. 1 *E* and *F* and Datasets S1 and S2), argues in favor of the more complete PSI structure as the predominant in vivo species.

We identified densities for 170 chlorophyll (Chl) *a*, 1 Chl *a*’, 13 Chl *b*, 46 carotenoids (Cars), 3 Fe_4_S_4_, 2 phylloquinones, and 56 bound lipid molecules in the *Ds*PSI-LHCI_1_ supercomplex (*SI Appendix*, Figs. S7 and S8 and Table S2). Although *D. tertiolecta* and *D. salina* are separated by ~253 million years of evolution and inhabit distinct ecological niches, coastal Arctic vs. hypersaline desert, the PSI-LHCI supercomplexes from the Fe-replete condition are highly similar, underscoring the high conservation of PSI subunit organization and antenna arrangement in the *Dunaliella* radiation (Fig. 2). For *D. tertiolecta*, we identified 167 Chl *a*, 1 Chl *a*’, 13 Chl *b*, 42 Car, 3 Fe_4_S_4_, 2 phylloquinones, and 42 lipid molecules (*SI Appendix*, Figs. S7 and S9 and Table S2). The poor resolution of the *D. tertiolecta* PSAH1 and PSAL1 subunits precludes identification of associated pigments, Chl *a*201, and a carotenoid of PSAH1 as well as Chl *a*302, Chl *a*305, and a carotenoid of PSAL1 (*SI Appendix*, Table S2).

### An Additional, TIDI1-Containing, LHCI Tetramer in Fe-Starved Dunaliella PSI-LHCI Supercomplexes.

Cryo-EM analysis of the PSI-LHCI supercomplexes from Fe-starved cells revealed major populations (~60% and 30% for *D. salina* and *D. tertiolecta,* respectively) distinct from the PSI-LHCI_1_ structures found in the Fe-replete cells. The resulting structures (hereafter named PSI–LHCI_2_) reached overall resolutions of 2.9 Å for *D. salina* and 2.7 Å for *D. tertiolecta*. The PSI–LHCI_2_ supercomplexes have an additional crescent-shaped LHCI tetramer with associated pigments that is evident in both *Dunaliella* spp. grown in Fe-starved conditions (Fig. 2 *B* and *D* and *SI Appendix*, Figs. S10–S13 and Table S3 and S4). Both *Dunaliella* spp. PSI–LHCI_2_ supercomplexes contain 10 PSA subunits, an inner LHCI tetramer arranged as in the PSI-LHCI_1_ supercomplex structure, and an outer LHCI tetramer (Fig. 2 *B* and *D* and *SI Appendix*, Table S3 and S4).

Like the PSI-LHCI_1_ supercomplexes, these PSI–LHCI_2_ supercomplex densities are more rigid around the PSI core and more flexible in the crescent-shaped LHCI tetramer regions, with the outer LHCI tetramer being even more flexible (Fig. 2 *B* and *D* and *SI Appendix*, Figs. S10–S12). Local refinement leading to a 3.3 Å global resolution for this region allowed us to identify LHCA1, LHCA8, and LHCA7, with the fourth subunit identified as TIDI1 after even further local refinement (*SI Appendix*, Figs. S14 and S15). The larger size of the *Ds*PSI–LHCI_2_ supercomplex versus the *Dt*PSI–LHCI_2_ supercomplex in the biochemical preparations can be explained, in part, by the presence of PSAG1, PSAH1, PSAO1, and the LHCA2-LHCA9 dimer in the *Ds*PSI–LHCI_2_ supercomplex, but not the *Dt*PSI–LHCI_2_ supercomplex (*SI Appendix*, Fig. S3). The biochemical preparations from Fe-starved *D. tertiolecta* cells contained a minor population of PSI-LHCI_1_ supercomplexes (~20%, resolved to 3.1 Å) containing PSAG1, LHCA2, and LHCA9 (*SI Appendix*, Fig. S11), indicating that the absence of these subunits in the *Dt*PSI–LHCI_2_ complex is not trivially explained by dissociation during sample preparation or vitrification.

In terms of pigments and cofactors, we identified *D. salina* and *D. tertiolecta* PSI–LHCI_2_ supercomplexes to contain, respectively, 218 and 185 Chl *a*, 23 and 20 Chl *b*, 59 and 47 Car, 56 and 26 lipids, plus 3 Fe_4_S_4_, 1 Chl *a*’, and 2 phylloquinones in the reaction center (*SI Appendix*, Figs. S7–S9 and Table S3 and S4). The majority of the pigment differences between *D. salina* and *D. tertiolecta* correspond to pigments associated with the three extra PSA and two extra LHCA subunits in the *Ds*PSI–LHCI_2_ supercomplex, accounting for a difference of 30 Chl *a*, 3 Chl *b,* and 11 Car molecules (*SI Appendix*, Table S3 and S4). We could not confidently model Chl *a*302, Chl *a*305, and a carotenoid in PSAL1 of the *Dt*PSI–LHCI_2_ supercomplex due to poor density in those regions.

Two crescent-shaped tetramers are a common feature in chlorophyte PSI-LHCI supercomplexes, as observed in *Chla. reinhardtii*, *Bryopsis corticulans*, and *Chlorella ohadii* (22–24). But the outer LHCI tetramer in *Dunaliella* spp. is distinct. First, a conventional LHCA protein is replaced by TIDI1, and second, its orientation within the supercomplex is unique (Fig. 2 *B* and *D*). The inner LHCI tetramer, containing LHCA1, LHCA8, LHCA7, and LHCA3, has a similar association with the PSI core in all reported PSI structures in the Viridiplantae clade (*SI Appendix*, Fig. S16 *A* and *B*) (18, 25). The outer LHCI tetramer of the *Dunaliella* spp. PSI–LHCI_2_ supercomplexes is rotated toward PSAK1 by ~33 degrees, whereas in other reported green algal structures, the outer LHCI tetramer is aligned with the inner LHCI tetramer (22–24) (*SI Appendix*, Fig. S16*A*). Outer LHCI tetramer rotation occurs also in a bryophyte (*Physcomitrium patens*) large PSI-LHCI supercomplex, but through a very distinct mechanism that involves association with additional LHCII oligomers (*SI Appendix*, Fig. S16*B*) (26, 27). In contrast, in *Dunaliella* spp., LHCA1 of the outer LHCI tetramer (hereafter LHCA1b) interacts with inner LHCI tetramer subunits (see below) that are closer to the PSAG1 side (Fig. 2 and *SI Appendix*, Fig. S16*B*). These distinct arrangements of the outer tetrameric LHCA subcomplexes in divergent organisms may represent independent convergent mechanisms for adjusting PSI accessory light-harvesting antenna.

The *Dunaliella* outer LHCI tetramer forms a crescent-shaped arrangement similar to the inner LHCI tetramer. Superimposition of the inner and outer LHCI tetramers results in a RMSD of 0.36 and 0.66 Å (over 223 and 224 aligned core Cα atoms) for *Ds*PSI–LHCI_2_ and *Dt*PSI–LHCI_2_ supercomplexes, respectively (*SI Appendix*, Fig. S17*A*). This suggests that the relative positions of each of the LHCA monomers are well conserved within the inner and outer LHCI tetramers. Interestingly, when the outer LHCI tetramers of *Ds*PSI–LHCI_2_ and *Dt*PSI–LHCI_2_ supercomplexes are superimposed, the RMSD is 0.56 Å (over 223 aligned core Cα atoms) (*SI Appendix*, Fig. S17*A*). This indicates the high conservation of the arrangement of LHCA proteins in the outer LHCI tetramer between two divergent *Dunaliella* spp. in Fe starvation. However, the outer LHCI tetramer of the PSI–LHCI_2_ supercomplexes is slightly less curved than the inner tetramer in both *Dunaliella* spp. (*SI Appendix*, Fig. S17*B*), which are similarly observed in other PSI-LHCI supercomplexes with a double LHCI tetramer antenna arrangement (23, 27).

**Fig. 3.**
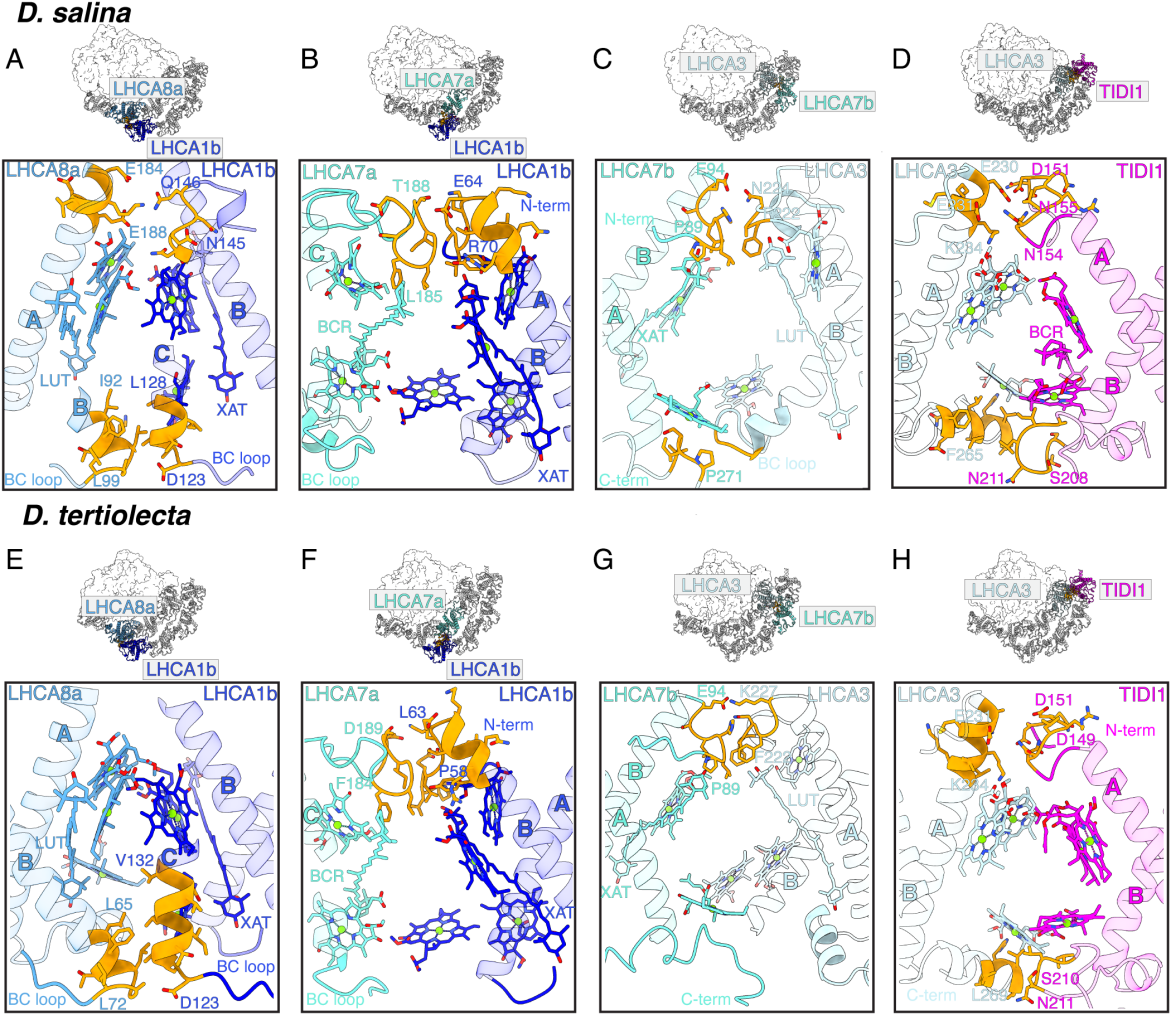
The interactions between the inner LHCI tetramer and the outer LHCI tetramer in the *Ds*PSI–LHCI_2_ and *Dt*PSI–LHCI_2_ supercomplexes. (*A*–*H*) *Top*: The interaction regions between the inner and outer LHCI tetramers in the overall *Ds*PSI–LHCI_2_ (*A*–*D*) and *Dt*PSI–LHCI_2_ (*E*–*H*) supercomplexes. Boxed areas are enlarged in the indicated panels. *Bottom*: protein–protein, pigment–pigment, and protein–pigment interactions between: LHCA8a and LHCA1b (*A* and *E*), LHCA7a and LHCA1b (*B* and *F*), LHCA3 and LHCA7b (*C* and *G*), LHCA3 and TIDI1 (*D* and *H*). The residues involved in the interactions are highlighted as yellow stick representations.

The intersubunit interactions in the inner and outer tetramer are also conserved in the two *Dunaliella* spp. (Fig. 3 and *SI Appendix*, Fig. S18). On the PSAG1 side, LHCA1b interacts with both LHCA8 and LHCA7 in the inner LHCA tetramer (hereafter LHCA8a and 7a) (Fig. 3 and *SI Appendix*, Fig. S18 *A* and *E*). The lumen side of helix C in LHCA1b interacts with the BC loop of LHCA8a through hydrophobic side chains, while its stromal side interacts with the AC loop of LHCA8a through charged side chains. The latter interaction is only evident in the *Ds*PSI–LHCI_2_ supercomplex but is poorly resolved in the *Dt*PSI–LHCI_2_ supercomplex (Fig. 3 and *SI Appendix*, Fig. S18 *A* and *E*). The N-terminus of LHCA1b uses hydrophobic side chains to interact with the AC loop of LHCA7a on the stromal side (Fig. 3 and *SI Appendix*, Fig. S18 *B* and *F*). At the PSAK1 side of the inner crescent, LHCA7 of the outer LHCI tetramer (hereafter named LHCA7b) interacts with LHCA3 through both polar and nonpolar side chains at its stromal side N-terminus (Fig. 3 and *SI Appendix*, Fig. S18 *C* and *G*). In the *Ds*PSI–LHCI_2_ structure, we note an interaction between the lumen side C-terminus of LHCA7b and the AC loop of LHCA3, but this is not well-resolved in our EM density of the *Dt*PSI–LHCI_2_ structure (Fig. 3 and *SI Appendix*, Fig. S18 *C* and *G*). Several pigments, including Chl and Car, and nonprotein densities attributed to lipids were observed in these interfaces, suggesting their contribution to the attachment of the outer and inner LHCI tetramers (*SI Appendix*, Fig. S7).

### TIDI1 Is a Key Constituent of the Second LHCI Tetramer.

We confirmed the location of TIDI1 as the most distal subunit in the outer LHCI tetramer of the PSI-LHCI_1_ supercomplex using TIDI1-specific features relative to LHCA3 and other LHCAs. These TIDI1-specific features include an extended BC loop with the diagnostic PFXGX_2_PF motif as well as primary sequence differences (*SI Appendix*, Figs. S19 *B* and *D*) (8, 28). However, the extended proline-rich region between L31 and S147 for *Ds*TIDI1 and L129 for *Dt*TIDI1 (8) was too flexible and could not be resolved. The presence of TIDI1 in the outer LHCI tetramer instead of LHCA3 is consistent with the reduced abundance of LHCA3 relative to LHCA1, LHCA7, and LHCA8 in whole-cell proteomics analysis (Fig. 1 *E* and *F*).

**Fig. 4.**
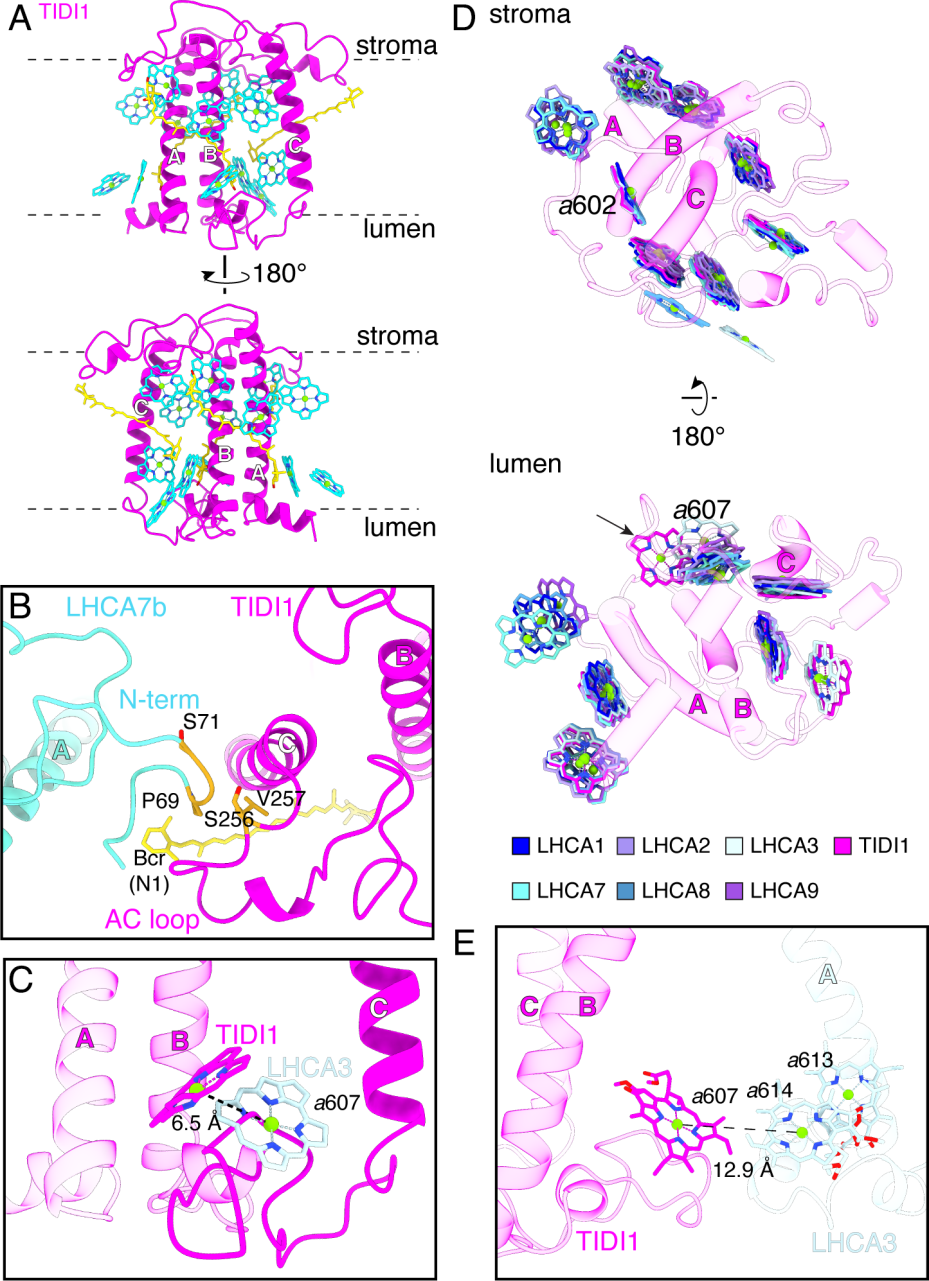
The extended BC loop of *Ds*TIDI1 differently positions Chl 607. (*A*) Cartoon representation of *Ds*TIDI1. Chls are shown as cyan stick representation, with the central Mg atoms shown as spheres and numbered according to the conserved sites in spinach LHCII (PDB: 1RWT). Carotenoids are shown as yellow-colored stick representation. (*B*) Interactions between *Ds*TIDI1 and *Ds*LHCA7b in the outer LHCI tetramer are colored in orange. Residues involved in the interaction are shown as orange sticks. (*C*) Comparison of Chl *a*607_TIDI1_ (magenta) position against position of *a*607_LHCA3_ (light blue) from *D. salina* on the lumenal side. Distance between molecules is shown as dashed lines. (*D*) Comparison of *Ds*TIDI1 Chl positions against Chl positions of *Ds*LHCA1, *Ds*LHCA2, *Ds*LHCA3, *Ds*LHCA7, *Ds*LHCA8, and *Ds*LHCA9 from *D. salina* on the stromal (*Top*) and lumenal side (*Bottom*). (*E*) Side view of *D. salina a*607_TIDI1_ (magenta) and its distance shown as dashed lines to *a*614_LHCA3_ (light blue).

Like other members of the LHC family, TIDI1 has three transmembrane helices (Fig. 4*A* and *SI Appendix*, Fig. S19*A*), and its interactions via its stromal AC loop with the N-terminus of the adjacent LHCA7b subunit are similar to the LHCA3–LHCA7a interaction (Fig. 4*B* and *SI Appendix*, Fig. S19*B* and S20 *A* and *C*). The TIDI1-specific BC loop contacts helix C of LHCA3 at the lumenal side in both *Dunaliella* spp. PSI–LHCI_2_ supercomplexes, with polar side chains contributing to the interaction (Fig. 3 and *SI Appendix*, Fig. S18 *D* and *H*). Charged side chains are also involved in the stromal side interaction between the N-terminus of TIDI1 and the AC loop of LHCA3, reminiscent of the interaction of the N-terminus of LHCA3 with the PsaA subunit for association of the inner-tetramer with the PSI core (Fig. 3 *D* and *H* and *SI Appendix*, Fig. S20 *B* and *D*). The stroma- and lumen-side TIDI1–LHCA3 interactions suggest a role for TIDI1 in positioning the outer LHCI tetramer with respect to the inner LHCI tetramer.

We modeled three Cars in TIDI1 in the same location as those in LHCA3 in both structures, and 13 Chls in *D. salina* and 12 in *D. tertiolecta* (Fig. 4*A* and *SI Appendix*, Fig. S19*A* and Tables S3 and S4). Chl *a*611 in *D. tertiolecta* could not be modeled because of poor density in that region. As in other LHC subunits, the Chl–Car interactions occur primarily at the stromal pigment layer, contributing to the asymmetric enrichment of pigments at the stromal side. All Chl positions of TIDI1 were equivalent to those in LHCA3, except for Chl *a*607 (hereafter *a*607_TIDI1_) on the lumenal side. Compared to *a*607_LHCA3_, *a*607_TIDI1_ is shifted 6.5 Å or 7.4 Å (Mg to Mg distance) and rotated by ~ 67 or 61 degrees in the *D. salina* and *D. tertiolecta* supercomplexes, respectively (Fig. 4*C* and *SI Appendix*, Fig. S19*C*). The position and orientation of *a*607_TIDI1_ are unique to TIDI1 and have not been observed in any other LHC protein (Fig. 4*D* and *SI Appendix*, Figs. S19*D* and S21). The TIDI1-specific extended BC loop, especially residues G219-I228 in *D. salina* and G204-I213 in *D. tertiolecta*, have an impact on *a*607_TIDI1_ orientation (*SI Appendix*, Figs. S8 and S9). *a*607_TIDI1_ is at the interface of TIDI1 and LHCA3 at the lumen side and is 12.9 Å and 12.5 Å (Mg to Mg distance in *D. salina* and *D. tertiolecta*, respectively) from *a*614_LHCA3_ (Fig. 4*E* and *SI Appendix*, Fig. S19*E*), close enough for energy transfer between TIDI1 and LHCA3. The similar positions and orientations of the majority of the pigments in TIDI1 to LHCA3 support its evolution from LHCA3 and functional equivalency in energy transfer.

### Multiple Evolutionary Paths to an Outer LHCI Tetramer.

The double LHCI tetramer arrangement occurs in two different ways (aligned versus severely rotated) in the Viridiplantae clade. In many chlorophyte algae, the expansion of the *LHCA* genes gave rise to LHCA4, LHCA5, and LHCA6 (29). LHCA5 and LHCA6 are specialized with extended lumenal C-terminal regions that facilitate the association of the outer and inner LHCI tetramers to form the aligned outer LHCI tetramer arrangement (22–24) (*SI Appendix*, Fig. S16*C*). When they are found in green algal genome (e.g. Chlamydomonas, *Bry. corticulans*, *Chlo. ohadii*, and *Coccomyxa subellipsoidea*), LHCA4, LHCA5, and LHCA6 co-occur, consistent with their cofunction and cooperation in forming the outer LHCI (22–24, 30). In *P. patens*, the subunits of the inner LHCI tetramer are reused in the outer LHCI tetramer to form a very tilted offset outer LHCI tetramer that connects also with LHCB9 and an LHCII trimer on the opposite side (27) (*SI Appendix*, Fig. S16*C*). Here, we identify a third arrangement in *Dunaliella* spp. where the outer LHCI tetramer is facultative. It is induced in Fe starvation by selective stoichiometric adjustment of three inner tetramer subunits (LHCA1, LHCA8, and LHCA7) along with the innovation of a unique LHCA-type protein (TIDI1) (Fig. 2 *B* and *D*), likely evolved from an ancestral LHCA3 (8). The result is a distinct LHCI tetramer whose orientation is also tilted and offset, albeit less so than in bryophytes (27). These comparisons underscore the relevance of the extended C-termini in the chlorophyte LHCA5 and LHCA6 for the aligned tetramer arrangements.

## Discussion

### Convergent Evolution of Modified PSI Antenna in Fe-Starved Algae.

Poor Fe bioavailability impacts primary productivity at a global scale (1, 2, 31) because of loss of the photosynthetic apparatus. At a biochemical level, PSI and ferredoxin are prime targets due to their abundance and high Fe content, and their reduced abundance is a classic biomarker for the Fe-starved state (4, 6, 32–36). A well-known adaptation, occurring in both cyanobacteria and some eukaryotic algae, is the replacement of iron-containing ferredoxin by iron-independent flavin-containing flavodoxin, which reduces the Fe quota of the cell, yet enables continued photosynthesis-driven reductive metabolism (8, 17, 36–39). Fld is reduced by electrons from PSI whose abundance and hence throughput is impacted by Fe starvation induced PSI retrenchment. In cyanobacteria, the *isiB* gene encoding Fld is coexpressed with *isiA*, which encodes a Chl-binding protein (also named CP43’ because of its sequence relationship to CP43 (PsbC) in PSII) (17, 40–42). Increased expression of *isiA* in Fe-starved cyanobacteria promotes remarkable reorganization and expansion of the PSI–antenna complex to compensate for the retrenchment of the PSI core complex (43, 44).

The coexpression of IsiA with IsiB is likely required to maximize anabolic metabolism in the Fe-starved cyanobacteria. A survey of 159 cyanobacterial genomes showed that 76 do contain the *isiA* gene and of those, the vast majority (71) also encode a Fld (*SI Appendix*, Fig. S22 and Dataset S3). In a majority (56) of the genomes that contain both, the *isiA* and *isiB* genes are adjacent and colinear, possibly in an *isiAB* operon as is the case in *Synechococcus elongatus* PCC 7942 (45). Fld is not present in streptophytes, but does occur in some green algae, including *Dunaliella* spp., and typically it occurs with a TIDI1-homolog (8, 46) (Fig. 1*B*). The co-occurrence of Fld and TIDI1 in diverse green algae [separated by ~650 million years (8)] evokes an adaptive strategy reminiscent of the cyanobacterial IsiA/IsiB (Fld) system. Introduction of Fld into crop plants as a strategy for increasing photosynthetic resilience has been discussed, but thus far it has not yielded significant improvements in low Fe situations (47). PSI antenna reorganization may be one approach to achieving this goal.

**Fig. 5.**
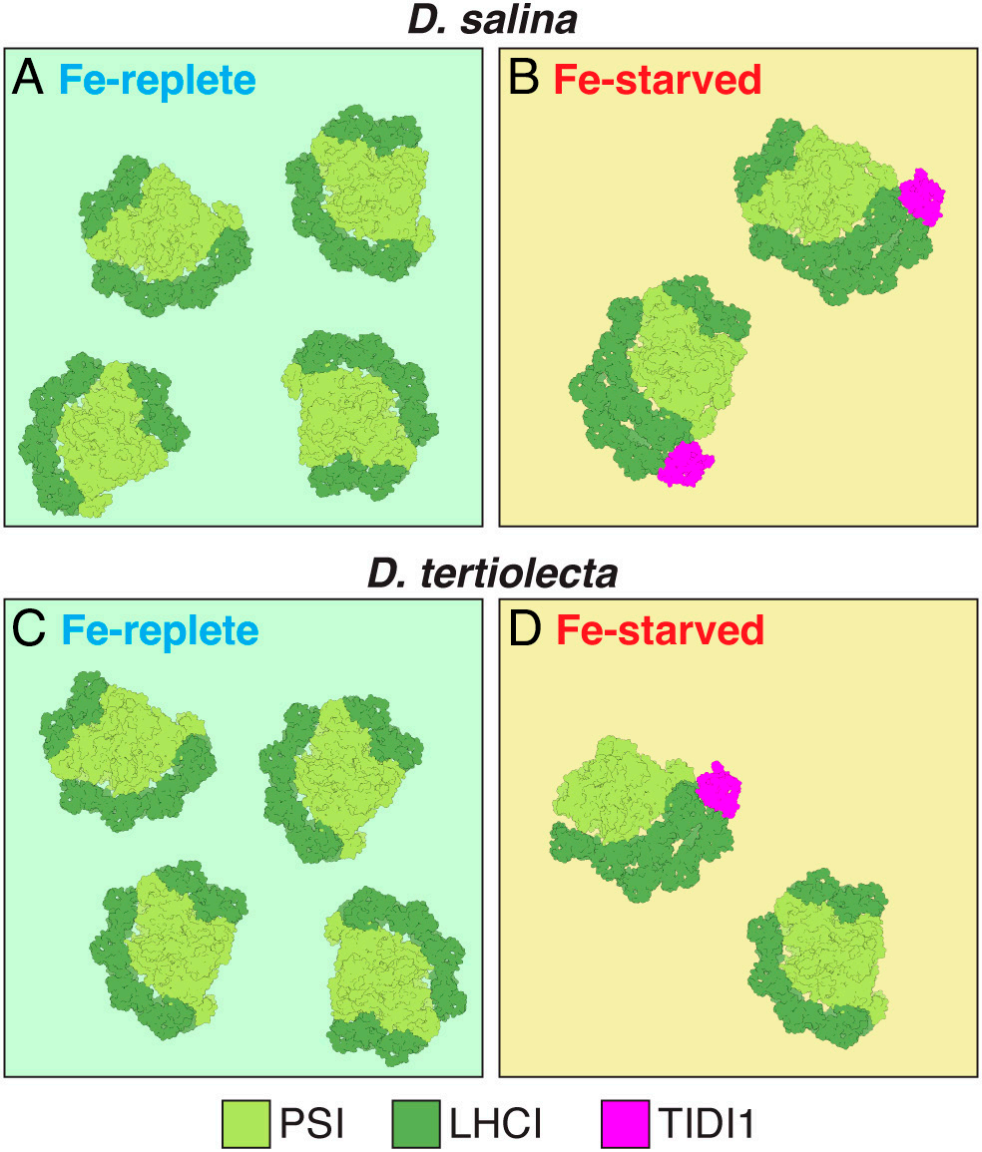
Fe-nutrition responsive modification of PSI-LHCI antenna in *Dunaliella* spp (*A*-*D*). (*A* and *C*) PSI-LHCI supercomplexes in Fe-replete cells have a single LHCI tetramer (dark green) composed of four subunits in *D. salina* (*A*) and *D. tertiolecta* (*C*). When cells become Fe-starved, the PSI-LHCI supercomplexes have an additional LHCI tetramer (dark green) also composed of four subunits with the novel, Fe starvation–induced chlorophyll-binding protein, TIDI1 (pink), in *D. salina* (*B*) and *D. tertiolecta* (*D*). The PSI core subunits are colored in light green. The *Dt*PSI–LHCI_2_ does not include the PSAO1, PSAG1, PSAH1, LHCA2, and LHCA9 subunits when compared to those from *D. salina* cells. Fe-starved *D. tertiolecta* samples include a small fraction of *Dt*PSI–LHCI_1_ supercomplexes, as shown.

The metabolic adaptations in play in eukaryotes have not been well investigated beyond documentation of PSI structural and functional reduction (48–50). Here, we fill this gap by structural analysis of PSI-LHCI supercomplexes from ~253 million years’ divergent *Dunaliella* spp. from distinct saline environments, Arctic water (*D. tertiolecta*), and desert hypersaline lake (*D. salina*) that exhibit unusual productivity with low Fe supplementation (*SI Appendix*, Fig. S1) (8). We find that increased expression of TIDI1 under Fe starvation is correlated with the formation of a novel TIDI1-containing LHCI tetramer that associates with the PSI-LHCI supercomplex to effect a larger antenna analogous to the long known rearrangement of the prokaryotic PSI antenna (8, 14, 43, 44) (summarized in Fig. 5). We showed that PSI subunit abundances decrease substantially to effect a lower intracellular Fe quota (Fig. 1 and *SI Appendix*, Fig. S1). The larger PSI antenna of the remaining Dunaliella PSI-LHCI supercomplexes perhaps allows maintenance of PSI photochemistry during Fe starvation for balanced operation of the photosynthetic apparatus.

### Fe starvation Induced Antenna Diversity in other Eukaryotic Algae.

The distinctive structural rearrangement of the LHCI antenna in Fe-starved *Dunaliella* spp. was foreshadowed by whole-cell proteomic analyses (Fig. 1 *D* and *E*), which revealed uniform heterogeneity in both species in the response of three of the six LHCA subunits during the Fe starvation-dependent retrenchment of the photosynthetic apparatus. LHCA1, LHCA7, and LHCA8, which are constituents of the novel outer LHCI tetramer, were retained, but LHCA2 and LHCA9, which make up the dimer and LHCA3 that connects the tetramer to the PSI core, decreased in proportion to the decrease in PSI core subunits. The structures of the PSI–LHCI_2_ supercomplex explain the discrepancy because three of the four subunits of the inner tetramer are reused in the new TIDI1-containing tetramer (Fig. 2 *B* and *D*). Whole-cell proteomics is a valuable tool for assessing the physiological relevance of unusual subunit compositions in isolated complexes. Although we did not undertake absolute quantification of each of the subunits in the Fe-replete cells, we note that PSAG1, PSAL1, PSAH1, PSAI1, LHCA2, and LHCA9 were captured in the proteomics dataset of both species (Fig. 1 *E* and *F* and Dataset S2), and indeed these subunits were found in both Fe-replete structures (Fig. 2 *A* and *C*). We did not observe particles corresponding to the minimal PSI-LHCI supercomplex found in other work with *D. salina* (21), and we wonder whether subunit dissociation may occasionally be a problem during isolation or vitrification. Interestingly, most of the same subunits (PSAG1, PSAH1, LHCA2, and LHCA9) are absent from the *Dt*PSI–LHCI_2_ supercomplex in our present work as well.

Nevertheless, within the *D. tertiolecta* PSI-LHCI supercomplex preparations from Fe-starved cells, we found the Fe-replete-type PSI-LHCI_1_ supercomplexes with the typical PSA and LHCA subunit compositions, including PSAG1, PSAH1, LHCA2, and LHCA9 (Fig. 2*D* and *SI Appendix*, Fig. S11). This finding indicates that the isolation and vitrification of the *D. tertiolecta* PSI-LHCI supercomplexes from the Fe-starved cells were not especially harsh. Yet, these subunits are absent in the TIDI1-containing *Dt*PSI–LHCI_2_ supercomplex. Given that the proteomics data indicate similar relative protein abundance changes in *D. tertiolecta* and *D. salina* for the PSA and LHCA subunits (Fig. 1 *E* and *F*), it is possible that the LHCA2 and LHCA9 subunits are preferentially lost from the *Dt*PSI–LHCI_2_ supercomplex (51). It is also possible that the absence of LHCA2, LHCA9, PSAG1, and PSAH1 may reflect genuine in vivo heterogeneity, which is not evident in the whole-cell proteomics because of the significant amount of the *Dt*PSI–LHCI_1_ supercomplexes in the preparation from Fe-starved cells (*SI Appendix*, Figs. S11 and S12). The net increase in Chl within the antennae of the Dunaliella PSI–LHCI_2_ supercomplexes is still between ~36 to 68% depending on retention of the LHCA2 and LHCA9 subunits. Even this amount of increase in the absorption cross-section can offer an incremental advantage that gets fixed through natural selection in environments under chronic Fe limitation.

The differential response of a subset of the LHCA proteins in *Dunaliella* spp. was distinct from observations of the well-studied chlorophyte *Chla. reinhardtii,* where all the subunits of the supercomplex are proportionally decreased under Fe-limited growth (*SI Appendix*, Fig. S23*B*) (52, 53). *Chla. reinhardtii* and *Chro. zofingiensis* have nine distinct and orthologous LHCA subunits versus six LHCA subunits in *Dunaliella* spp. (8, 17, 54). Although the structure of the *Cz*PSI-LHCI supercomplex from Fe-replete cells has not been determined, it is likely to be similar to that of the other chlorophyte algae (discussed above) with two aligned tetramers, an inner one made up of LHCA1, LHCA8, LHCA7, and LHCA3 and an outer one made up of LHCA1, LHCA4, LHCA5, and LHCA6 (22, 24). Whole-cell proteomics of *Chro. zofingiensis*, whose genome also encodes both FLD1 and TIDI1 (which are coordinately upregulated in Fe deficiency) (*SI Appendix*, Fig. S23*A*), also revealed selective differences in retention of some LHCA proteins (LHCA1, LHCA8, LHCA7, LHCA4, LHCA5, LHCA6) and proportional (to PSA subunits) losses of others (*SI Appendix*, Fig. S23) (55). We anticipate that structural analysis of the Fe-starved PSI-LHCI supercomplex from *Chro. zofingiensis* will reveal another novel quaternary arrangement of LHCI tetramers.

In contrast, there are a few green algae, like *Coccomyxa subellipsoidea*, whose genomes encode a FLD1 ortholog and nine LHCA subunits but no TIDI1 ortholog (Fig. 1*B*)(56). A recent PSI-LHCI supercomplex structure of *Coc. subellipsoidea* revealed aligned inner and outer LHCI tetramers with LHCA4, LHCA5, and LHCA6 in the outer tetramer (30). It would be instructive to assess the resilience of such algae to variations in Fe nutrition with concomitant assessment of the structural organization of their PSI-LHCI supercomplexes. Another adaptation may be found in the diatom *Phaeodactylum tricornutum*, which has a FLD1 ortholog. The mRNA abundances for four Chl-binding proteins are upregulated in Fe-limitation, suggesting that there may be remodeling of a diatom-specific PSI-LHCI supercomplex (35, 36).

### Assembly of a Second LHCI Tetramer.

For both *D. tertiolecta* and *D. salina*, the arrangement of the PSI core and the inner LHCI tetramer subunits in the PSI–LHCI_2_ supercomplexes is almost identical to that in the PSI-LHCI_1_ supercomplexes (Fig. 2 and *SI Appendix*, Fig. S17). This observation is compatible with a model in which the PSI–LHCI_2_ supercomplex is assembled by the addition of the new tetramer to the preexisting PSI-LHCI_1_ supercomplexes. De novo synthesis of the entire supercomplex is unlikely, given that *PSA* transcript abundances are decreased under Fe starvation (8). The replacement of LHCA3 with TIDI1 may determine the position of the new tetramer on the outside rather than in direct association with the PSI core. Although we made a substantial effort to resolve the extended proline-rich region of TIDI1, including the use of a GraFix strategy for Fe-starved *D. tertiolecta*, we cannot assess its role in determining subunit–subunit interactions that promote assembly. The interaction of LHCA3 with the PSI core complex may be a common target for posttranslational events affecting PSI-LHCI function. In *Chla. reinhardtii*, the N terminus of LHCA3 is removed in low Fe, perhaps initiating the subsequent degradation of LHCA3 (57).

Together with the introduction of flavodoxin (58), eukaryotic LHCI antenna remodeling presents a new engineering opportunity for improving PSI performance under low Fe nutrition stress, and in so doing can improve crop and oceanic productivity in environments that suffer from chronic Fe limitation.

## Materials and Methods

### Strains and Culture Conditions.

*Dunaliella tertiolecta* and *Dunaliella salina* were provided by Uri Pick. The medium composition and growth conditions were modified from (59). The NaCl concentration was reduced to 0.5 M, and the solution was de-metalated by passage through a Chelex 100 Resin (BioRad) column prepared according to (60). Fe was added to a final concentration of 15 or 0.15 µM FeCl_3_ with 6 µM EDTA, for the Fe-replete or the Fe-depleted conditions, respectively. All glassware was washed in 6 N HCl and rinsed several times with Milli-Q water. Cultures (100 to 500 mL) were grown in 250 mL to 1 L Fernbach flasks on shaking platforms (140 rpm) at 24 °C. Continuous light was provided by cool white fluorescent bulbs (4,100 K) and warm white fluorescent bulbs (3,000 K) in a 2:1 ratio at 100 µmol photons m^−2^ s^−1^. Cells were counted with a Beckman Coulter Multisizer 3 with a 50-µM orifice (Beckman Coulter).

### Proteomic Analyses.

Cells (1 × 10^8^) were collected by centrifugation at 1,680×*g* at 4 °C for 5 min and resuspended in 300 µL 10 mM sodium-phosphate, pH 7.0, frozen, and shipped for analysis at Environmental Molecular Sciences Laboratory (EMSL). The cell samples were fractionated and labeled with tandem mass tags as published in (61). Protein identities and abundances were quantified from all LC-MS/MS spectra using the software MSGF+ (62), and reporter ion abundances were extracted from each spectrum using MASIC (63) to measure peptide abundance. Each peptide abundance was normalized to the mean central tendency of all peptide abundances per sample. Downstream statistical analysis of three separate cultures was conducted on log_2_ abundance of each sample. The protein names for *Chla. reinhardtii* and *Dunaliella* spp. are based on the updated nomenclature from (54).

### Isolation of PSI-LHCI Supercomplexes.

*Dunaliella* Fe-starved and Fe-replete thylakoid membranes were first purified by sucrose cushion centrifugation as described previously (26) with modifications. Cultures containing 1 to 3 × 10^6^ cells/mL were collected at 1,670×*g* for 5 min at 4 ºC (JLA-10.500 rotor, Beckman Coulter). Cells were washed once (*D. tertiolecta*) or twice (*D. salina*) in a growth medium (described above) containing the respective Fe concentrations. The cell pellet was osmotically lysed on ice for 30 min in 25 mM MES-NaOH (pH 6.5), 1.5 mM NaCl, 0.2 mM benzamidine, and 1 mM ε-aminocaproic acid with intermittent gentle agitation.

To isolate the PSI-LHCI supercomplex, thylakoid membranes (400 to 800 µg Chl at 0.5 mg Chl/mL) were solubilized with 1% (w/v) *n*-dodecyl-α-D-maltoside (α-DDM) (Anatrace) for 30 min with gentle agitation. The unsolubilized membranes were removed by centrifugation at 20,000×*g* for 5 min at 4 °C. The proteins from the solubilized membranes were separated by density gradient centrifugation containing either a sucrose gradient [0.1 to 1.8 M sucrose with 25 mM MES-NaOH (pH 6.5) and 0.03% (w/v) α-DDM] for negative staining or maltose gradient for cryo-EM data collection [0.1 to 1.4 M maltose with 25 mM MES-NaOH (pH 6.5) and 0.03% (w/v) α-DDM at 154,300×*g* (SW 41 Ti rotor, Beckman Coulter) for 24 h at 4 °C. PSI-LHCI supercomplexes were collected dropwise from the bottom of the tube before negative staining or single-particle cryo-EM analysis.

To stabilize the complexes further including TIDI1, mild crosslinking using the GraFix procedure (28) was performed for a second round of cryo-EM data collection for Fe-starved *D. tertiolecta*. For GraFix, a gradient was prepared by mixing the light (0.1 M maltose with 25 mM MES-NaOH (pH 6.5) and 0.03% (w/v) α-DDM) and heavy solutions (1.4 M maltose with 25 mM MES-NaOH (pH 6.5), 0.03% (w/v) α-DDM, and 0.075% glutaraldehyde (v/v)). The proteins from the solubilized thylakoid membranes were both separated and gently crosslinked with the GraFix technique, and the centrifugation was carried out as described earlier at 154,300 x*g* (SW 41 Ti rotor, Beckman Coulter) for 24 h at 4 °C. The crosslinking reaction in the GraFix purified supercomplexes was quenched with 100 mM MES-Tris-HCl (pH 6.5).

### Cryo-EM Sample Preparation and Data Collection.

Cryo-EM grids were prepared with in-house fabricated carbon on gold grids, Quantifoil Au/Cu R1.2/1.3 grids 300 mesh, or Quantifoil Au/Cu R2/1 grids 300 mesh. The grids were washed with chloroform and layered with graphene oxide as previously described (64). Briefly, 4 µL of 1 mg/mL of polyethylenimine HCl MAX Linear MW 40 k (PEI, Polysciences) in 25 mM HEPES-NaOH (pH 7.5) was applied and incubated on glow discharged grids for 2 min, blotted away, and washed twice with 4 µL water. The PEI-treated grids were dried with Whatman paper. 0.2 mg/ml of graphene oxide (Sigma-Aldrich, 763705) prepared in 1:5 methanol: Water solution was vortexed for 10 s and precipitated at 1,000×*g* for 1 min. 4 µL of the supernatant was applied to the PEI- treated grids for 2 min, blotted away, washed twice with 4 µL water, and dried for at least 15 min before freezing grids.

Samples were diluted with filtered 25 mM MES-NaOH (pH 6.5), 0.02% (w/v) α-DDM and 0.5% trehalose (w/v) to optimize particle distribution on the grid. Glow-discharged graphene oxide layered grids (64) (glow discharged for 30 s, 15 W, 64 (N/255), 10 sccm, and no purging) were mounted in a Vitrobot Mark IV (Thermo Fisher Scientific) maintained at 10 °C and 100% relative humidity, with lights off. 4 µL of diluted sample was incubated for 1 min on the grid before blotting (blot time 7 to 9 s and blot force 7) and immediately plunge-frozen in liquid ethane.

Frozen grids were loaded onto a Titan Krios G3i microscope (Thermo Fisher Scientific) operated at an acceleration voltage of 300 keV, equipped with a Gatan K3 summit direct electron detector operated in CDS mode, and with a GIF Quantum energy filter with a 20 eV slit width. Data collection was carried out at a nominal magnification of 81,000× in superresolution mode (superresolution pixel size of 0.525 Å per pixel) and with a defocus range of –0.8 µm to –2.0 µm. The total electron exposure was about 55 to 60 e/Å^2^ and each video stack comprised 50 frames. The data collection was set up using SerialEM and monitored using CryoSPARC Live (65). The same data collection strategy was used for all our cryo-EM datasets. 5371 movies were collected for Fe-replete *D. salina* samples, 5044 movies for Fe-replete *D. tertiolecta* samples, 6,270 movies for Fe-starved *D. salina* samples, 11,299 movies for Fe-starved *D. tertiolecta*, and 11,529 movies for Fe-starved *D. tertiolecta* isolated with a GraFix strategy were collected.

### Electron Microscopy Data Processing.

All movies were aligned, gain corrected, and binned by two using MotionCorr2 implemented in RELION (v.3.0) (66). Contrast transfer function was computed with CTFFIND4 implemented in RELION (v.3.0). Particles were picked using crYOLO (v.1.7.7.1) with a box size of 280 pixels (67). Particle coordinates from crYOLO were imported into RELION3, extracted with a box size of 240 pixels (binned by two), and subjected to one round of 2D classification. Good classes that represented different views of PSI-LHCI supercomplexes were selected (326,845 particles from Fe-replete *D. salina* samples, 522,452 particles from Fe-replete *D. tertiolecta* samples, 190,745 particles from Fe-starved *D. salina* samples, and 518,929 particles from Fe-starved *D. tertiolecta*).

For the Fe-replete *D. salina* and *D. tertiolecta* datasets, particles from good 2D classes underwent a round of 3D classification using a low-pass filtered map generated from PDB 6SL5 as an initial reference and classified into three classes. The 3D class with the highest resolution was selected for iterative rounds of 3D and CTF refinement, resulting in a final global resolution of 2.8 Å for Fe-replete *D. salina* PSI-LHCI_1_ supercomplexes and 2.1 Å for Fe-replete *D. tertiolecta* PSI-LHCI_1_ supercomplexes.

Particles from good 2D classes of Fe-starved *D. salina* dataset underwent a round of 3D classification using a low-pass filtered map generated from PDB 6SL5 (20) as an initial reference and classified into three classes. The particles from two 3D classes that had a second LHCI tetramer were combined, resulting in 120,321 particles. These particles underwent two rounds of 3D refinement that resulted in a 3D reconstruction at final global resolution of 3.0 Å. To improve the density of the PSAO1 and TIDI1 subunits, PSAO1 and TIDI1-specific masks were used for particle subtraction. The subtracted particles underwent focused classification without alignment, resulting in a final global resolution of 3.3 Å for the PSAO1 mask and TIDI1 mask.

As for Fe-starved *D. salina* dataset, particles from 2D classes from the Fe-starved *D. tertiolecta* dataset underwent a round of 3D classification using a low-pass filtered map generated from PDB 6SL5 as an initial reference and classified into three classes. The 3D class with a second LHCI tetramer had 123,335 particles and that with a single LHCI tetramer had 108,975 particles. These particles were then subjected to iterative rounds of 3D and CTF refinement, which resulted in a final map at 2.7 Å global resolution for the PSI–LHCI_2_ supercomplex and 3.1 Å for PSI-LHCI_1_ supercomplex. To improve the density of the LHCA1 subunit, an LHCA1-specific mask was used for particle subtraction. The subtracted particles underwent focused classification without alignment resulting in a final map at 3.3 Å global resolution for the LHCA1.

The selected particles from Fe-starved *D. tertiolecta* dataset obtained using GraFix underwent 3D classification using a low-pass filtered map generated from PDB 6SL5 as an initial reference and classified into 4 classes. The 3D class with a second LHCI tetramer contained 217,073 particles. These particles then underwent iterative rounds of 3D and CTF refinements which resulted in a final map at 2.8 Å global resolution for the double LHCI tetramer structure. To improve the density for the PSAL1 and TIDI1 subunits, PSAL1 and TIDI1-specific masks were used for particle subtraction. The subtracted particles underwent focused refinement without alignment, resulting in a final map at 3.2 Å for the PSAL1 mask and TIDI1 mask.

The workflows for all the datasets are described in *SI Appendix*, Figs. S4, S5, and S10–S12.

### Model Building and Validation.

The atomic models for the PSI structures were built by fitting the existing structure of *D. salina* (PDB identifier 6SL5) (20) as a template using PHENIX v.1.21.1 dock in map and subsequently refined with PHENIX real-space refinement (68). The amino acid sequences were then mutated to match the *D. salina* and *D. tertiolecta* genome and rebuilt on the basis of the cryo-EM density using COOT v.0.9.8.7 (8, 69). The initial TIDI1 model was built using AlphaFold2 from the TIDI1 sequences from either *D. salina* or *D. tertiolecta*, docked into the Fe-starved *Dunaliella* PSI–LHCI_2_ complexes using PHENIX dock in map, and subsequently fitted for the side chains in COOT (70). Models were built sequentially, starting with the maps of the refined structure, then the locally refined regions (PSAG1, PSAH1, PSAL1, PSAK1, PSAO1, LHCA1, LHCA2, and the outer tetramer), and finally the TIDI1 density. The resulting atomic models were refined using both the refined map and locally refined maps with the PHENIX real space refinement program using restraints for Chl *a* from (71) and the Grade Web Server (https://grade.globalphasing.org/cgi-bin/grade2_server.cgi) for other ligands. This process was followed by manual inspection to remove obvious errors. The refinement statistics are provided in Extended *SI Appendix*, Table S1. Figures were prepared using UCSF ChimeraX v.1.7.1 (72). Interactions between subunits were analyzed through the PDBe PISA server (https://www.ebi.ac.uk/msd-srv/prot_int/cgi-bin/piserver) (73). The protein names for *Chla. reinhardtii* and *Dunaliella* spp. are based on the updated nomenclature from (54).

## Supplementary Material

Appendix 01 (PDF)

Dataset S01 (XLSX)

Dataset S02 (XLSX)

Dataset S03 (XLSX)

## Data Availability

EM maps and models, raw micrographs, and proteomics data have been deposited in RCSB Protein Data Bank, Electron Microscopy Data Bank, Electron Microscopy Public Image Archive, Center for Computational Mass Spectrometry (9MH0, 9MGW, 9MH1, 9MGZ, EMD-48266, EMD-48264, EMD-48265, EMD-48262, EMPIAR-12655, EMPIAR-12653, EMPIAR-12656, EMPIAR-12654, MSV000096479, MSV000096475, MSV000096362). The atomic coordinates for *D. salina* have been deposited in the PDB with the accession codes 9MH0 (74) for the Fe-replete PSI-LHCI_1_ supercomplex and 9MGW (75) for the Fe-starved PSI–LHCI_2_ supercomplex. The atomic coordinates for *D. tertiolecta* have been deposited in the PDB with the accession codes 9MH1 (76) for the Fe-replete PSI-LHCI_1_ supercomplex and 9MGZ (77) for the Fe-starved PSI-LHC_2_ supercomplex. The electron microscopy maps have been deposited in the Electron Microscopy Data bank with the accession codes 48266 (78) for *Dt*PSI-LHC_1_ supercomplex, 48264 (79) for the *Dt*PSI-LHC_2_ supercomplex, 48265 (80) for the *Ds*PSI-LHC_1_ supercomplex, and 48262 (81) for the *Ds*PSI-LHC_2_ supercomplex. The raw micrographs for all datasets have been deposited in the Electron Microscopy Public Image Archive with the accession code EMPIAR-12655 (82) for the *Dt*PSI–LHCI supercomplexes from Fe-replete cells, EMPIAR-12653 (83) for the *Dt*PSI–LHCI supercomplexes from Fe-starved cells, EMPIAR-12656 (84) for the *Ds*PSI-LHCI supercomplexes from Fe-replete cells, and EMPIAR-12654 (85) for the *Ds*PSI-LHCI supercomplexes from Fe-starved cells. The proteomics data utilized for this study have been deposited in the Center for Computational Mass Spectrometry with the accession codes MSV000096479 for *D. salina* and *D. tertiolecta (55)*, MSV000096475 for *Chla. reinhardtii (54)*, and MSV000096362 for *Chro. zofingiensis (57).* All other data are included in the manuscript and/or *SI Appendix*.
