# Supplementary material for: A distinct LHCI arrangement is recruited to photosystem I in Fe-starved green algae
Source: Proc Natl Acad Sci U S A. 2025 Jun 16;122(25):e2500621122. doi: 10.1073/pnas.2500621122 (PMC12207447; doi:10.1073/pnas.2500621122)
Supplement: Supplementary file 1 — Appendix 01 (PDF) [file pnas.2500621122.sapp.pdf]

PNAS

Supplemental Information for

A distinct LHCI arrangement is recruited to photosystem I in Fe-starved green algae

Helen W. Liu[1,†], Radhika Khera[1,2,†], Patricia Grob[2,3], Sean D. Gallaher[3], Samuel O. Purvine[4], Carrie D. Nicora[4], Mary S. Lipton[4], Krishna K. Niyogi[1,2,5], Eva Nogales[2,3,5,6], Masakazu Iwai[1,5,b,*], Sabeeha S. Merchant[1,3,6,7,a,*]

[1]Department of Plant and Microbial Biology, University of California, Berkeley, CA 94720, USA
[2]Howard Hughes Medical Institute, University of California, Berkeley, CA 94720, USA
[3]California Institute for Quantitative Biosciences (QB3), University of California, Berkeley, CA 94720, USA
[4]Earth and Biological Sciences Division, Pacific Northwest National Laboratory, Richland, WA 99352, USA
[5]Molecular Biophysics and Integrated Bioimaging Division, Lawrence Berkeley National Laboratory, Berkeley, CA 94720, USA
[6]Department of Molecular and Cell Biology, University of California, Berkeley, CA 94720, USA
[7]Environmental Genomics and Systems Biology, Lawrence Berkeley National Laboratory, Berkeley, USA, CA 94720

[†]H.W. Liu and R. Khera made equal contributions to this work.

Corresponding Authors (*): [a]Sabeeha S. Merchant and [b]Masakazu Iwai

Email: sabeeha@berkeley.edu and miwai@lbl.gov

This PDF file includes:

Fig. S1 to S23
Tables S1 to S5
Supplemental Materials and Methods
SI References

Supporting Methods

Elemental analysis

Elemental analysis was determined by ICP-MS/MS (1) with minor modifications. *Dunaliella* cells (6 x 10^6 cells/mL) from logarithmic growth (between 1 and 2 x 10^6 cells/mL) were collected by centrifugation at 1,680 x*g* for 3 min in a 50-mL Falcon tube. Cells were washed twice in a solution containing 1 mM Na_2-EDTA, pH 8, 0.5 M Chelex-treated NaCl and twice in Chelex-treated 0.5 M NaCl and collected by centrifugation. The cell pellet was digested with 143 µL of 70% nitric acid (Optima grade, Fisher, A467-500) at 65 ºC for 2–4 hours and diluted to a final nitric acid concentration of 2% (v/v) with Milli-Q water. Elemental analysis was by ICP-MS/MS on an Agilent 8900 Triple Quadrupole instrument, in comparison with an environmental calibration standard (Agilent 5183-4688), a sulfur (Inorganic Ventures CGS1) and a phosphorus standard (Inorganic Ventures CGP1). ^{89}Y (Inorganic Ventures MSY-100PPM) was used as an internal standard. The levels of all analytes were determined in MS/MS mode.

Chl content

Chl content was collected from 1 mL of culture (1–2 x 10^6 cells/mL) by centrifugation at 21,130 x*g* for 1 min at 4 °C and extracted with 80:20 (v/v) acetone to water. Debris was removed centrifugation at 21,130 x*g* for 5 min at 25 °C and the absorbance of the supernatant was measured at 647 nm and 664 nm to calculate Chl according to the extinction-coefficients shown previously (2). For isolated membranes, Chl was extracted from 5 µL of the sample (see below).

Preparation of TIDI1 *antiserum*

Affinity-purified rabbit polyclonal antibodies specific for TIDI1 were raised at Labcorp (Madison, Wisconsin, USA) against the peptide NH_2-PEPKKGSAFKGY-COOH (3) according to their protocols.

Immunodetection

10 mL of cultures at a density of 1–2 x 10^6 cells/mL was collected by centrifugation at 1,680 x*g* at 4 ºC and stored –80ºC. Cell pellets were resuspended in 300 µL 10 mM sodium phosphate, pH 7.0. Cells were broken by freeze-thaw cycling according to (4). Protein concentrations were determined by Pierce BCA assay against a bovine serum albumin standard (Thermo Fisher Scientific). Proteins were separated by SDS-PAGE and immunodetection as described by (4) except a different transfer buffer (25 mM Tris, 192 mM glycine, 20% (v/v) methanol) was used and TBS (10 mM Tris-HCl, 150 mM NaCl, pH 7.5) with 0.05% (w/v) Tween 20 (TBS-T) was used to dilute the antibodies and wash the membranes. Primary antibody dilutions were as follows: CF_1 1:50,000 (5), FDX1 1:500 (6), FLV1 1:500 (7), PSAF1 1:1000 (8), TIDI1 1:1000 (this publication). For visualization of bound antibody, washed membranes were incubated in a 1:6000 dilution of goat anti-rabbit secondary antibody (Southern Biotech) conjugated to alkaline phosphatase in 3% (w/v) nonfat dried milk in 1xPBS with 0.1% (w/v) Tween 20 and visualized by incubation for 0.5–1 min in 10 mL alkaline phosphatase buffer.

Fluorescence emission spectroscopy at 77K

Fractionated membranes or cultured cells at the mid-log phase were placed in a glass tube and frozen in liquid N_2 after adjusting the Chl concentrations to 5 µg/mL. Fluorescence emission was recorded at 77 K using FluoroMax-4 spectrophotometer (Horiba Scientific) according to the previous protocol (9).

Negative staining electron microscopy

The sample quality of the isolated PSI-LHCI supercomplexes was assessed through negative staining electron microscopy. Briefly, 4 µL of PSI-LHCI supercomplex samples, diluted in 25 mM MES-NaOH (pH 6.5) and 0.03% (w/v) α-DDM, was incubated for 1 min on carbon-coated Cu grids (400 mesh, Electron Microscopy Sciences) pretreated with a Tergeo-EM plasma cleaner (PIE

Scientific). The grid was subsequently washed with 2% (w/v) trehalose, 0.03% (w/v) α-DDM, and in 25 mM MES-NaOH (pH 6.5) and finally stained with three 50 µL drops of 1% (w/v) uranyl formate (SPI Supplies) before subsequent blotting and drying. Negatively stained samples were imaged in a Tecnai T12 electron microscope (Thermo Fisher Scientific), operated at 120 kV, 30,000x magnification using a TemCam F-416 camera (TVIPS).

isiA and fldA co-occurrence in Cyanobacteria

In order to identify the prevalence and co-occurrence of *isiA* genes encoding iron-stress-induced Chl-binding protein (CP43') and *fldA* (or *isiB*) genes encoding flavodoxin in Cyanobacteria, a systematic survey was conducted of the annotated Cyanobacterial genomes hosted in the National Center for Biotechnology Information (NCBI) genome database (ncbi.nlm.nih.gov). All genomes and proteomes within the taxon Cyanobacteriota (blue-green bacteria) that were described in that database as "reference genomes" were downloaded for analysis (N = 219). Three genomes described only as "cyanobacterium endosymbiont" were excluded, and three non-reference genomes (*Synechococcus* sp. PCC 7942, *Synechococcus* sp. PCC 7002, and *Synechocystis* sp. PCC 6803) were added based on their identification in Jia et al. (10). To exclude low quality, fragmented, or potentially non-axenic genome assemblies from further analysis, those with a CheckM completeness score <90%, a CheckM contamination score >10% or a contig N50 value <50 kb, as reported in the NCBI database were excluded. These filters left 156 high-quality Cyanobacterial genomes for further analysis.

A multispecies IsiA protein sequence (accession WP_041443458.1) was used as the query sequence in a blastp search with a minimum bit score of 250 to identify candidate IsiA proteins in the proteomes of the156 species identified above. IsiA belongs to a family of proteins that also includes Prochlorophyte chlorophyll-binding proteins (PcbA, PcbB, and PcbC), and the Chl *a*-binding proteins of PSII, CP43 and CP47. CP43 proteins were excluded from the list of candidate IsiA proteins by the presence of an extended "E" loop (11), and CP47 genes were

excluded by virtue of their lower sequence similarity (bit scores <90). From the remaining 165 candidate IsiA proteins, we could not identify any pattern that could discriminate between IsiA and PcbABC proteins. This is consistent with the conclusions of a previous study, which proposed unifying the family of IsiA and Pcb proteins and renaming them as accessory chlorophyll-binding proteins (CBPs) (12).

Fld proteins were identified using a multispecies FldA protein sequence (accession WP_012306911.1) in a blastp search of the 156 species' proteomes using a minimum bit score of 100. After extensive manual curation, the candidate IsiA / Pcb / CBP proteins, the FldA / IsiB proteins, and their corresponding gene and species data were compiled into a table (Dataset S3). For species with both *isiA* and *fldA* genes, the distance between genes in each species' genome assembly was calculated with in-house scripts. Genes were considered colinear if their loci were within 5 kb of each other on the same strand of the same contig. An Euler plot demonstrating the overlap of the presence of *isiA* and *fldA* genes in each species was constructed using the eulerr package in R.

Supporting figures

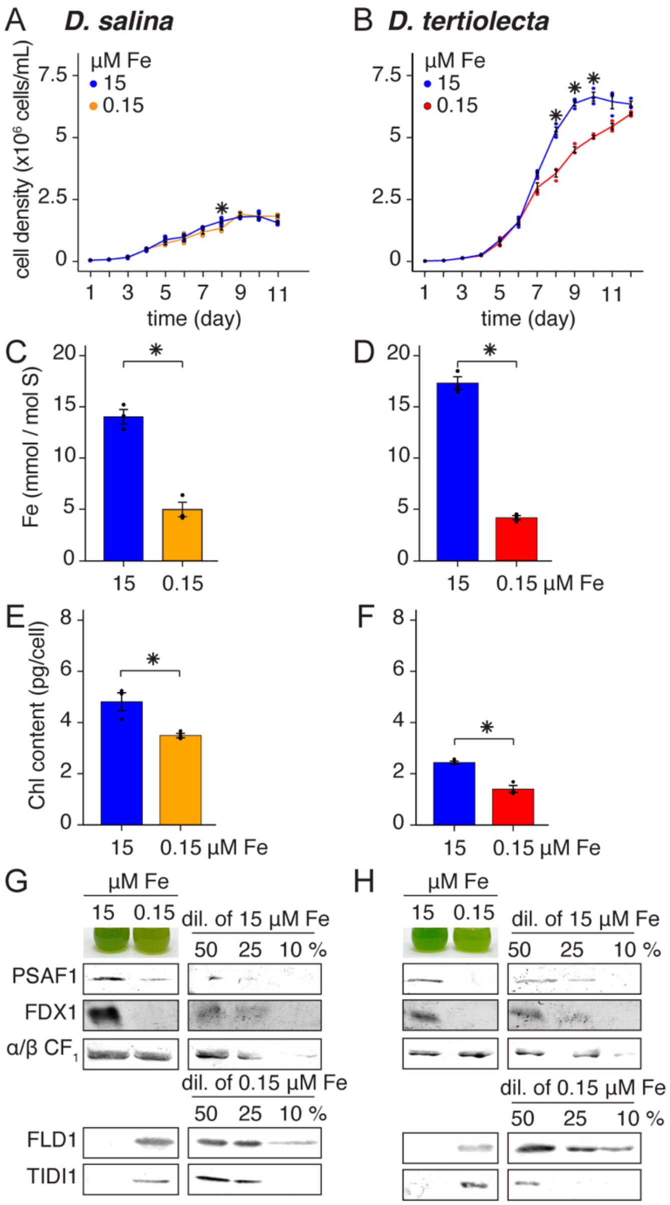

Fig. S1. *Dunaliella* spp. are resilient to Fe starvation. (A and B) Growth, (C and D) Fe content, (E and F) Chl content, and (G and H) immunodetection of photosynthetic proteins in *Dunaliella salina* (left) and *Dunaliella tertiolecta* (right) grown in Fe-replete (blue) and Fe-depleted (orange for *D. salina*, red for *D. tertiolecta*) medium. Standard error based on three independent cultures. *Asterisk* (*) denotes statistically significant difference relative to 15 µM Fe (p value < 0.05).

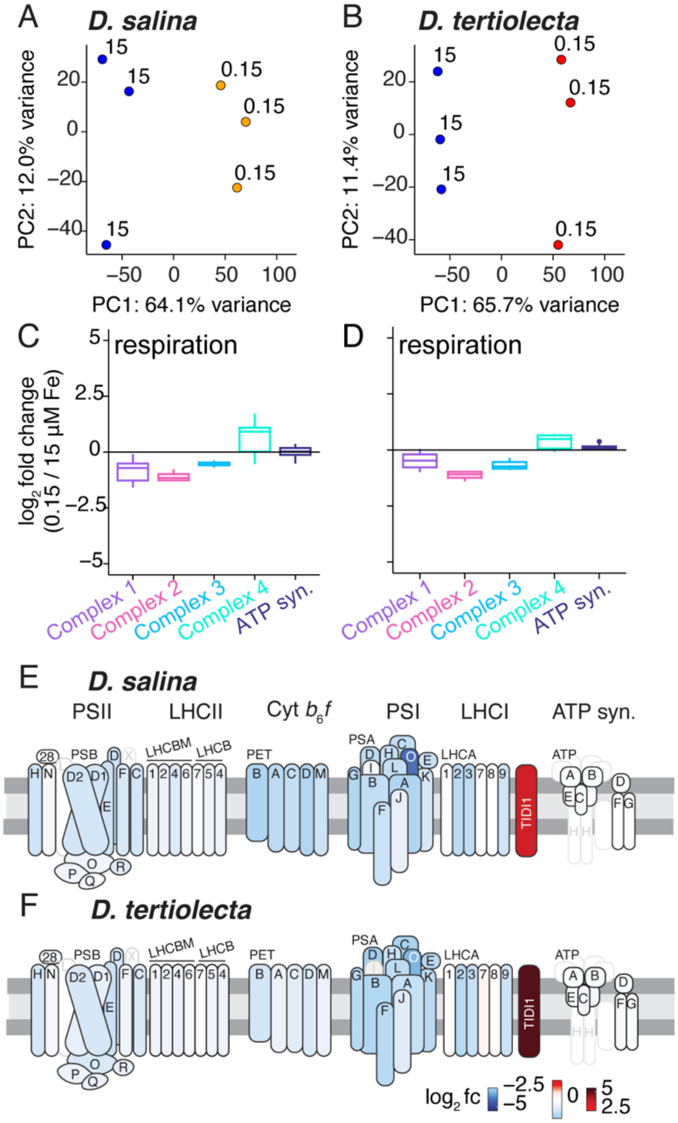

Fig. S2. The *Dunaliella* proteome responds to Fe-depletion. (A and B) The principal component analysis (PCA) separates the proteome of both *D. salina* (A) and *D. tertiolecta* (B) based on Fe availability (15 µM versus 0.15 µM medium). (C and D) The log_2 fold change of components of respiration (Complex 1, Complex 2, Complex 3, Complex 4, and ATP synthase). The full list of proteins included in the analysis is found in Dataset S2. (E and F) Overview of changes in abundance of proteins of the photosynthetic electron transfer change. Relative changes in protein abundances in Fe-starved (0.15 µM Fe) versus Fe-replete (15 µM Fe) medium as log_2 fold changes (red, increase; blue, decrease). Proteins are schematically assembled according to their position within the respective complex.

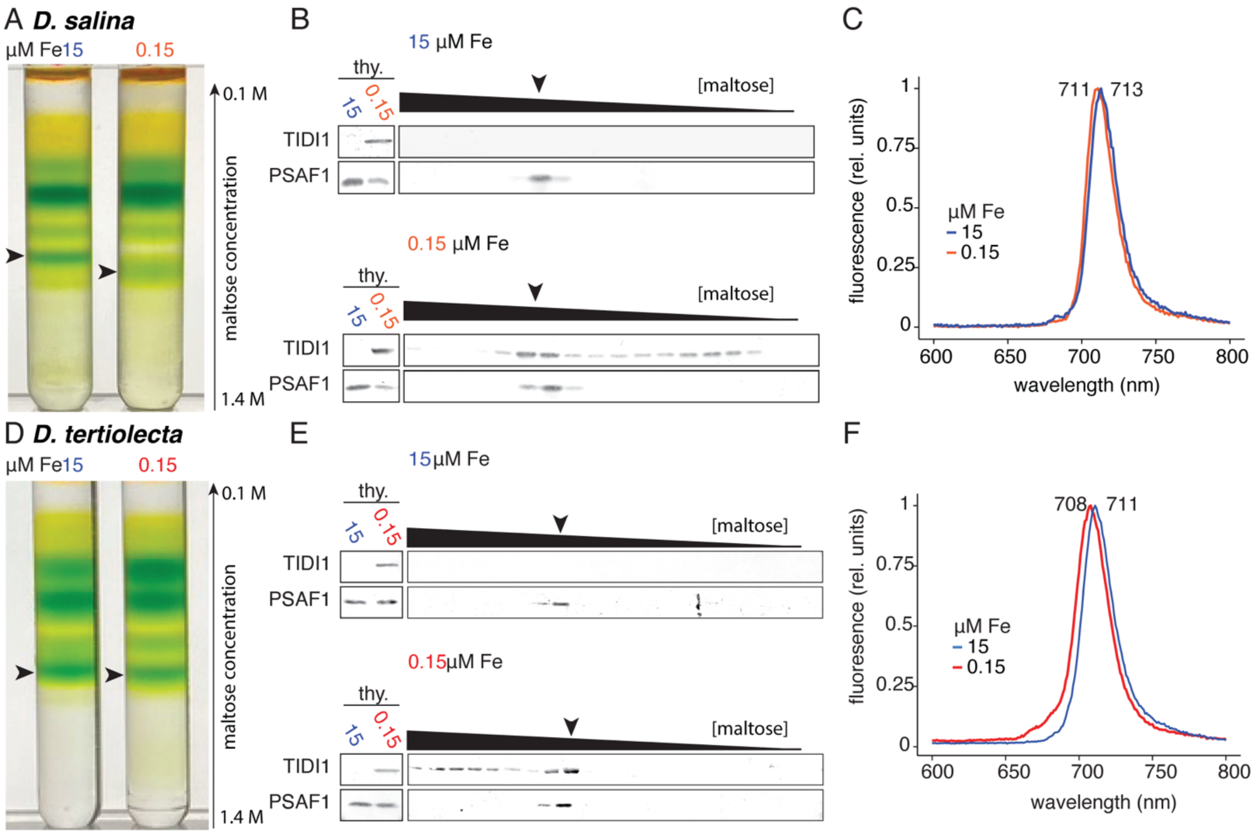

Fig. S3. Biochemical characterization of *Dunaliella* spp. PSI-LHCI supercomplex in Fe-replete and -starved conditions. (A and D) Isolation of PSI from thylakoid membranes of cells grown either in Fe-replete (left, 15 µM Fe) or -starved (right, 0.15 µM Fe) medium using maltose density gradient centrifugation for *D. salina* (A) and *D. tertiolecta* (D). Arrows indicate the locations of the bands corresponding to PSI. A representative result was shown from three independent thylakoid membrane isolations. (B and E) Immunoblot analysis showing the presence of PSI and TIDI1 in the thylakoids (thy.) (left) and location of PSI and TIDI1 within the maltose density gradient (right) from cells grown in either 15 µM Fe (top) or 0.15 µM Fe (bottom) medium for *D. salina* (B) and *D. tertiolecta* (E). (C and F) 77 K chlorophyll fluorescence emission spectra of isolated PSI isolated from cells grown 15 µM Fe (blue) or 0.15 µM Fe (orange) medium for *D. salina* (C) and *D. tertiolecta* (F).

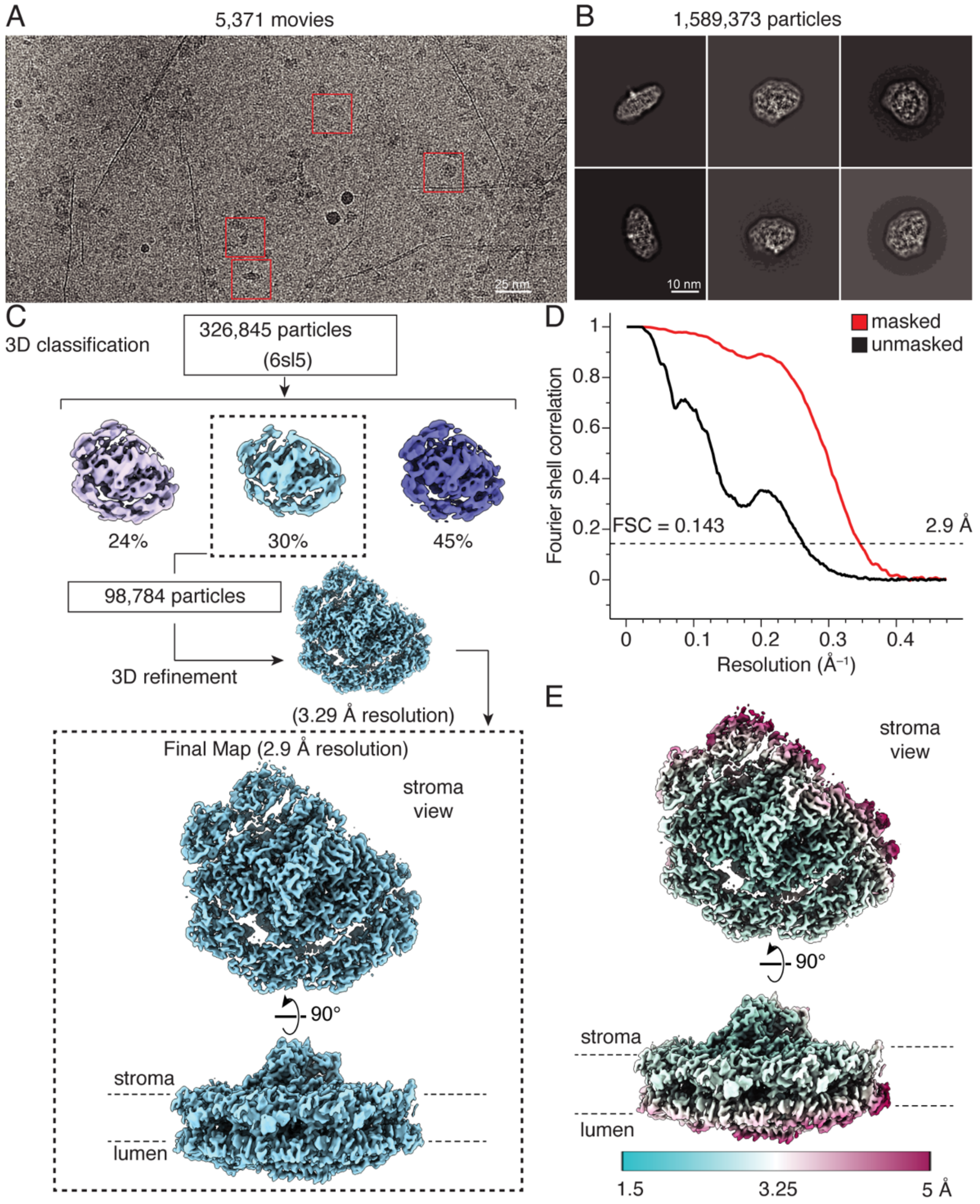

Fig. S4. Processing flowchart for the *D. salina* PSI-LHCI supercomplex in Fe-replete conditions. (A) Representative cryo-EM image of *D. salina* Fe-replete PSI-LHCI supercomplexes. (B) Representative 2D class averages of *D. salina* Fe-replete PSI-LHCI supercomplexes. Box size is 240 Å. (C) Processing flowchart for the cryo-EM dataset using RELION (v.3.0). (D) FSC curve of the final reconstruction. The profiles for masked and unmasked are red and black, respectively. (E) Local resolution for the final cryo-EM map.

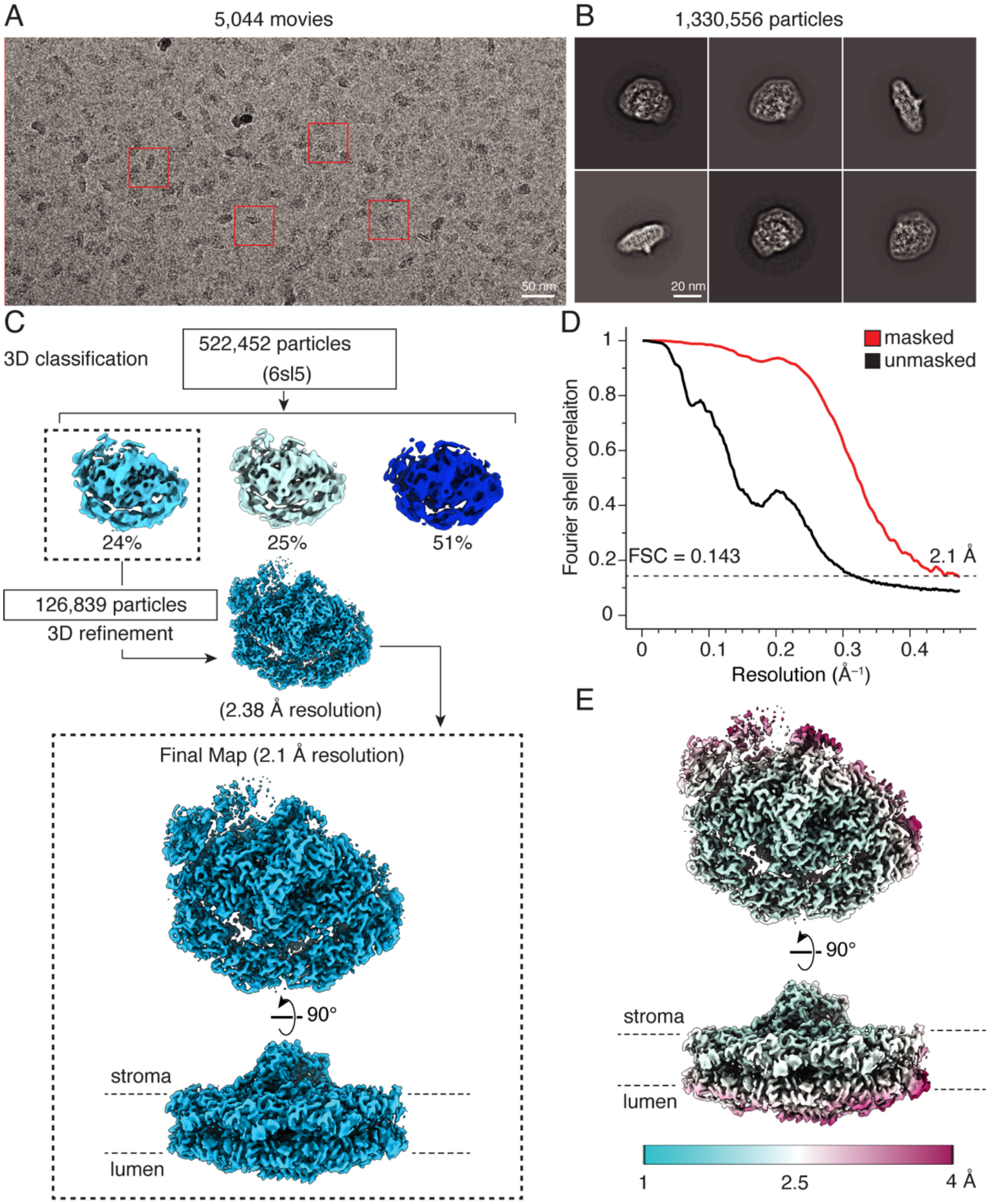

Fig. S5. Processing flowchart for the *D. tertiolecta* PSI-LHCI supercomplex in Fe-replete conditions. (A) Representative cryo-EM image of *D. tertiolecta* Fe-replete PSI-LHCI supercomplexes. (B) Representative 2D class averages of *D. tertiolecta* Fe-replete PSI-LHCI supercomplexes. Box size is 240 Å. (C) Processing flowchart for the cryo-EM dataset with RELION (v.3.0). (D) FSC curve of the final reconstruction. The profiles for masked and unmasked are red and black, respectively. (E) Local resolution for the final cryo-EM map.

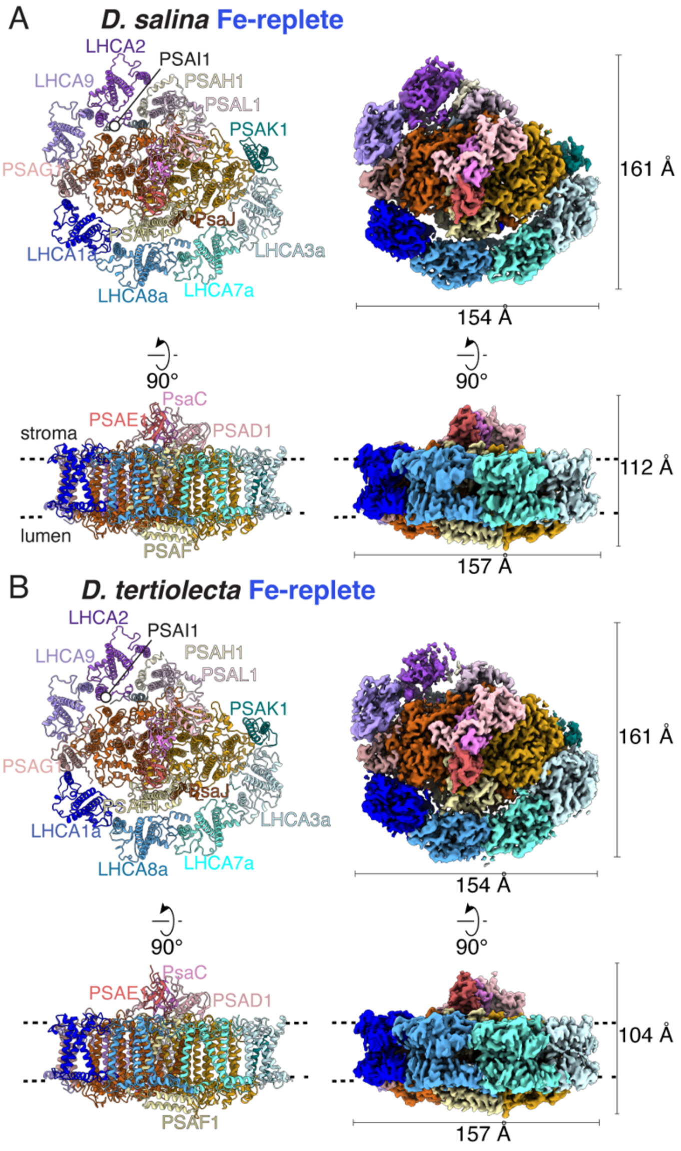

Fig. S6. Structural overview of the *Dunaliella* spp. PSI-LHCI$_1$ supercomplexes from Fe-replete cells. (A and B) The model (left) and the cryo-EM density maps (right) for PSI-LHCI$_1$ supercomplexes from Fe-replete *Dunaliella salina* (A) and *Dunaliella tertiolecta* (B).

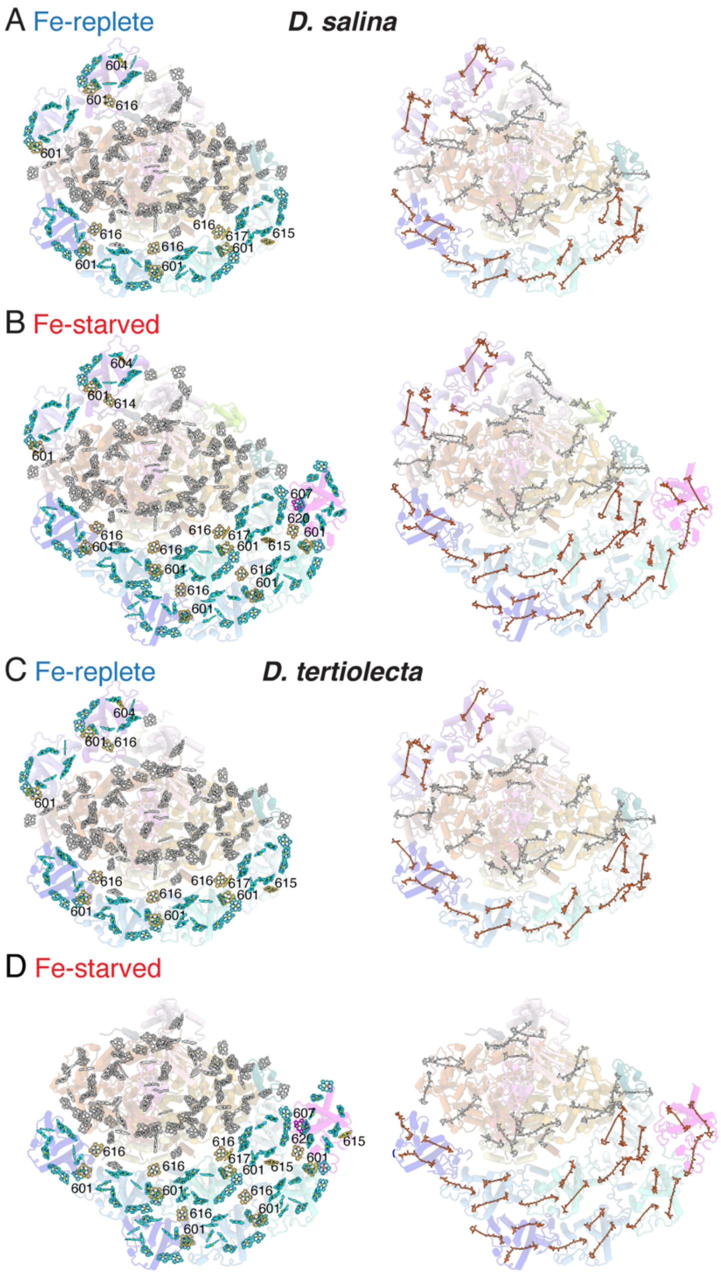

Fig. S7. Pigment arrangement in the *Ds*PSI-LHCI and *Dt*PSI-LHCI supercomplexes. (A-D) Left: Chl arrangement in *Ds*PSI-LHCI$_1$ (A), *Ds*PSI-LHCI$_2$ (B), *Dt*PSI-LHCI$_1$ (C) and *Dt*PSI-LHCI$_2$ (D) supercomplexes. Chls in the PSI core are colored in gray. Conserved Chl sites 601-614 are colored in cyan, and specific Chl sites 613-620 are colored in orange. (A-D) Right: Car arrangement in *Ds*PSI-LHCI$_1$ (A), *Ds*PSI-LHCI$_2$ (B), *Dt*PSI-LHCI$_1$ (C) and *Dt*PSI-LHCI$_2$ (D) supercomplexes. The PSI core Cars are colored in gray, and the Cars found in LHCA subunits are colored in red.

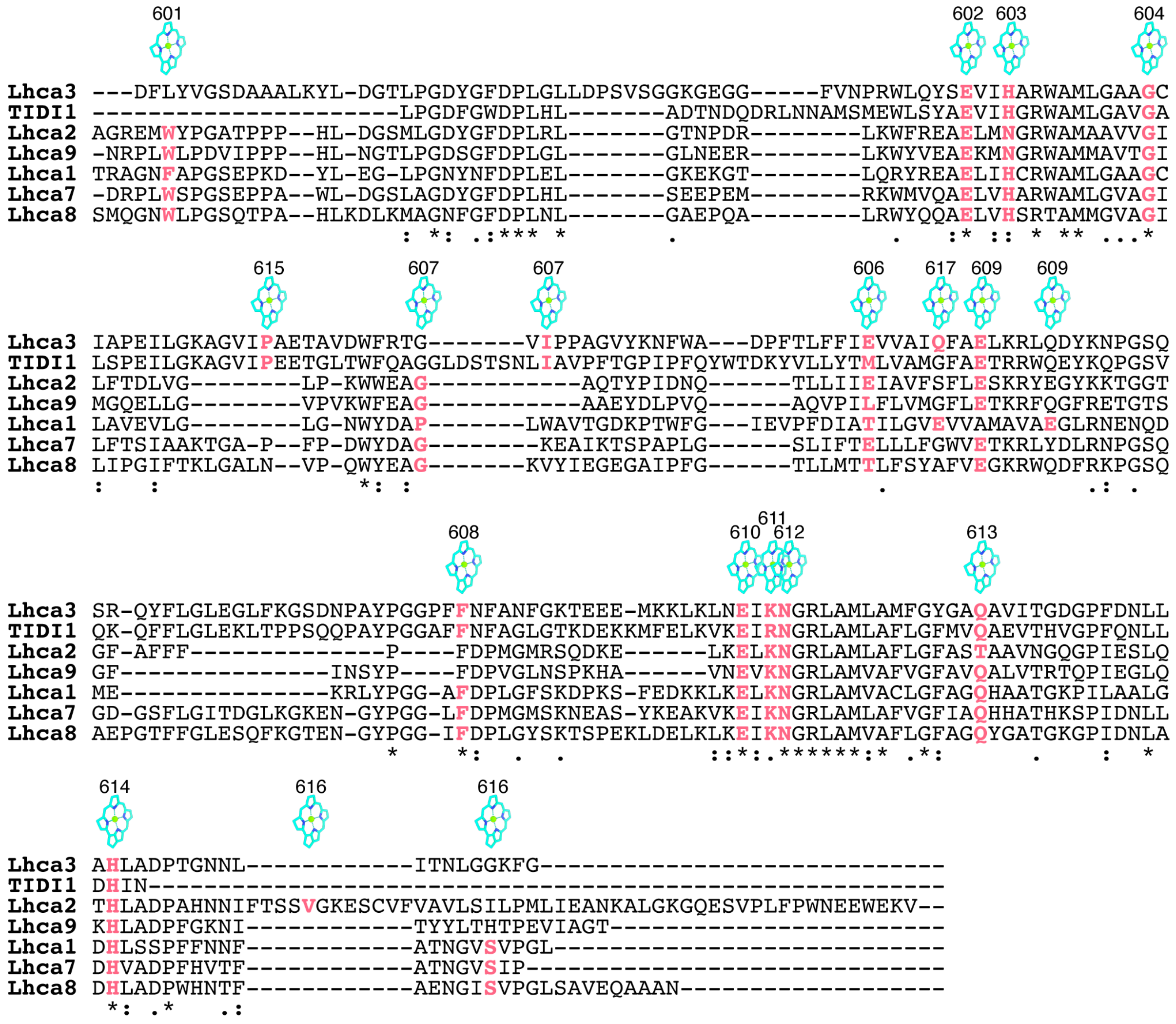

Fig. S8. Chl coordination of LHCI subunits from *D. salina* PSI-LHCI supercomplex. Multiple sequence alignment of LHCI subunits with residues involved in Chl coordination or contributing to Chl coordination are shaded in blue. Chl and their annotations are based on PDB:1RWT and shown above.

601 602 603 604

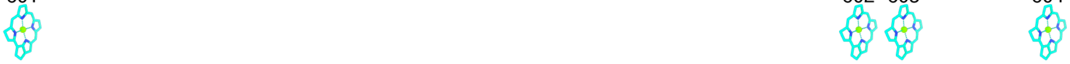

```
Lhca3 -----LYVGSDAAALKYL-DGTLPGDYGFDPLGLLDPSVSGGKGEGG-----FVNPRWLQYSEVIHARWAMLGAAGC
TIDI1 -------------------TGSLPGDFGWDPLHL-------ADTNDQDRLNNAMSMEWLSYAEVIHGRWAMLGAVGA
Lhca2 AAREMWYPGATPPA--HL-DGSMLGDYGFDPLRL-------GTNPDR--------MKWFREAELTNGRWAMAAVVGI
Lhca9 -NRPLWLPDVIPPP--HL-NGTLPGDSGFDPLGL-------GLNEER--------LKWYVEAEKMNGRWAMMAVTGI
Lhca1 QRAGNFAPGSEPKD--YL-EG-LPGNYNFDPLEL-------GKEKGT--------LQRYREAELIHCRWAMLGAAGC
Lhca7 ADRPLWSPGSEPPA--WL-DGSLAGDYGFDPLHL-------SEEPEM--------RKWMVQAELVHARWAMLGVAGI
Lhca8 NMNGNWLPGSQTPA--HLKDLKMAGNFGFDPLNL-------GAEPQA--------LRWYQQAELVHSRTAMMGVAGI
                             : *: .:*** *       .               .   :* :: * ** ...*
```

615 607 607 606 617 609 609

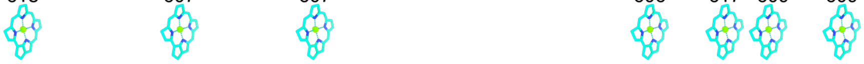

```
Lhca3 IAPEILGKAGVIPAETAVDWFRTG-------VIPPAGVYKNFWA---DPFTLFFIEVVAIQFAELKRLQDYKNPGSQ
TIDI1 LSPEILGKAGVIPEETGLTWFQAGGLDSTSNLIAVPFTGPIPFQYWTDKYVLLYTMLVAMGFAETRRWQEYKQPGSV
Lhca2 LFTDLVG--------LP-KWWEAG-----------AQTYPIDNQ------TLAIIEIAVFAFLEAKRYEGYKKTGGT
Lhca9 MGQELLG--------VPVKWFEAG-----------AAEYDLPVQ------AQVPILFLVMGFLETKRFQGFRETGTS
Lhca1 LAVEVLG--------LG-NWYDAP-------LWAVTGDKPTWFG---IEVPFDIATILGVEVVAMAVAEGLRNENQD
Lhca7 LFTSIAAKTGA-P--FP-DWYDAG--------KEAIKTSPAPLG------SLIFTELLLFGWVETKRLYDLRNPGSQ
Lhca8 LIPGIFTKLGALN--VP-QWYEAG-------KVYIEGEGAIPFG------TLLMTTLFSYAFVEGKRWQDFRKPGSQ
      :   :               *: :                             .              .: .
```

608 610 611 612 613

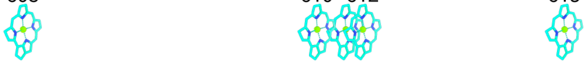

```
Lhca3 SR-QYFLGLEGLFKGSDNPAYPGGPFFNFANFGKTEEE-MKKLKLNEIKNGRLAMLAMFGYGAQAVITGDGPFDNLL
TIDI1 QK-QFFLGLEKLTPPSQQPAYPGGAFFNFAGLGTKDEKKMFELKVKEIRNGRLAMLAFLGFMVQAEVTHVGPFQNLL
Lhca2 GF-AFFF--------------P----FDPMGMRSPEKE------LKELKNGRLAMLAFLGFASTAAVNGQGPIESLQ
Lhca9 GF---------------INSYP----FDPVGLNSPKHA------VNEVKNGRLAMVAFVGFAVQALVTRTQPIEGLQ
Lhca1 ME---------------KRLYPGG-AFDPLGFSKDPKS-FEDKKLKELKNGRLAMVACLGFAGQHAATGKPILAALG
Lhca7 GD-GSFLGITDGLKGKEN-GYPGG-LFDPMGMSKNEAS-YKEAKVKEIKNGRLAMLAFVGFIAQHHATHKSPIDNLL
Lhca8 AEPGTFFGLESQFKGTEN-GYPGG-IFDPLGYSKTSPEKLDELKLKEIKNGRLAMVAFLGFAGQYGATGKGPIDNLA
                            *    *:  .  .          ::*:.******:* .*:      .    :  *
```

614 616 616

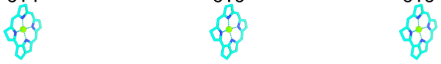

```
Lhca3 AHLADPTGNNL------------ITNLGGK-------------------------------
TIDI1 DHINSP-------------------------------------------------------
Lhca2 THLADPAHNNIFTSSVGKESCVFVAVLSVLPIIIEATKTLGKGESVPLFPWNEEWEKVA--
Lhca9 KHLADPFGKNI------------TYYLTHTPEVIAGT------------------------
Lhca1 DHLSSPFFNNF------------ATNGVSVPGV----------------------------
Lhca7 DHVADPFHVTF------------ATNGVSIPHFTEF-------------------------
Lhca8 DHLADPWHNTF------------AENGISVPGLSAVEQAAAS-------------------
       *: .*   .:
```

Fig. S9. Chl coordination of LHCI subunits from *D. tertiolecta* PSI-LHCI supercomplex. Multiple sequence alignment of LHCI subunits with residues involved in Chl coordination or contributing to Chl coordination are shaded in blue. Chl and their annotations are based on PDB:1RWT and shown above.

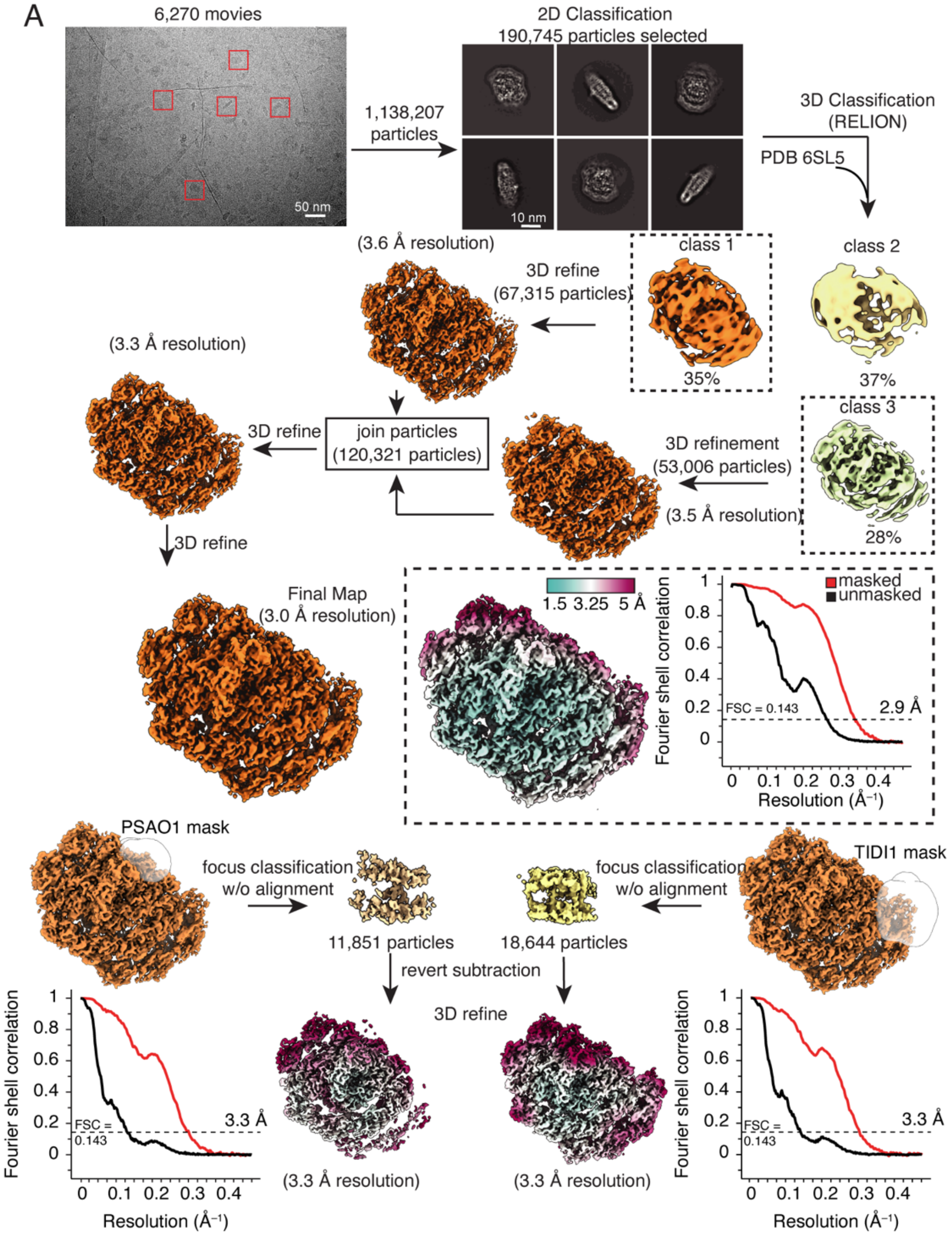

Fig. S10. Processing flowchart for *D. salina* PSI-LHCI supercomplex in Fe-starved conditions. (A) Flowchart showing all 3D classification and refinement steps used to determine the overall Fe-starved *D. salina* PSI-LHCI supercomplex using RELION (v.3.0).

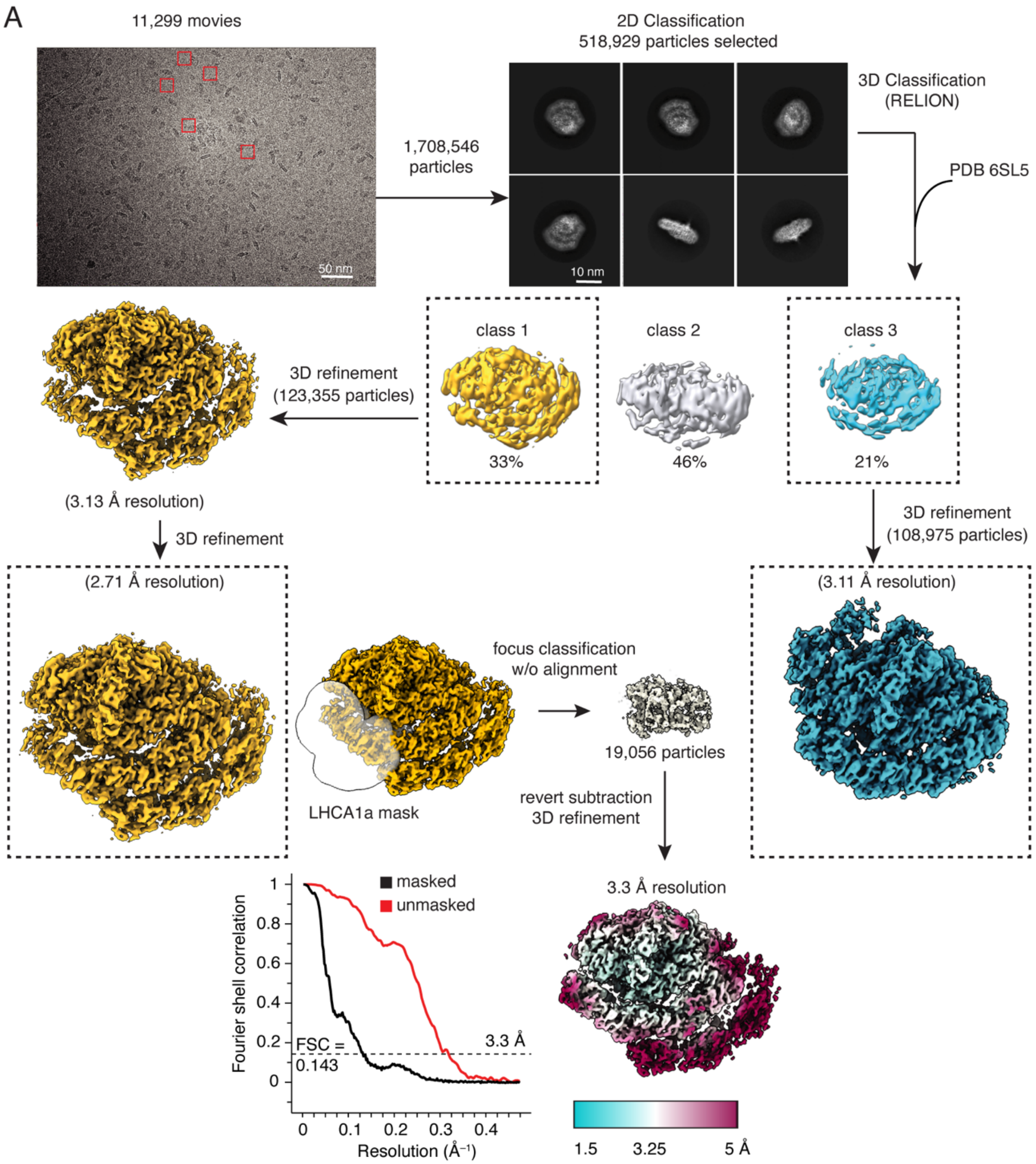

Fig. S11. Processing flowchart for *D. tertiolecta* PSI-LHCI supercomplex in Fe-starved conditions. (A) Flowchart showing all 3D classification and refinement steps used to determine the overall Fe-starved *D. tertiolecta* PSI-LHCI supercomplex using RELION (v.3.0).

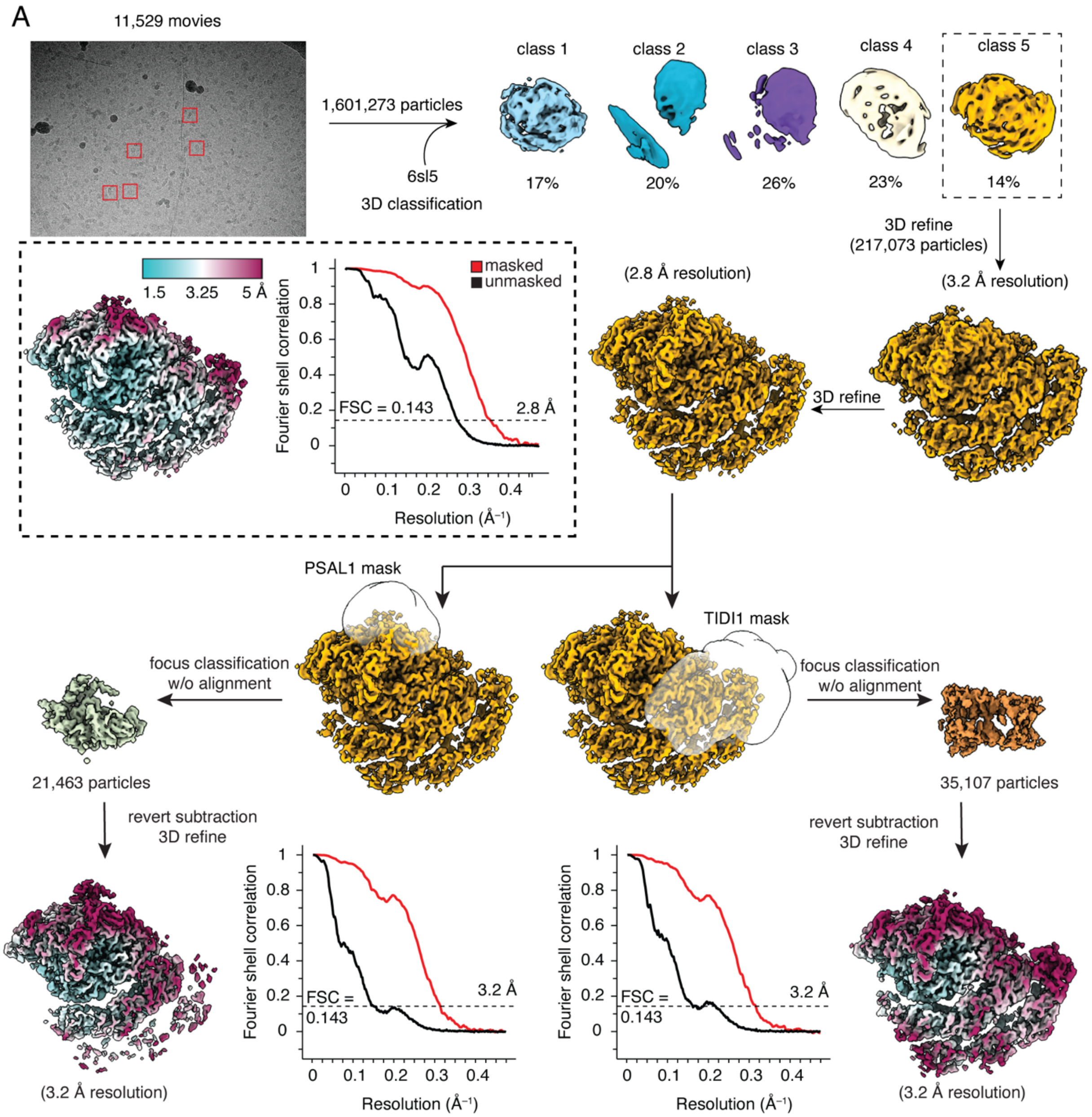

Fig. S12. Processing flowchart for *D. tertiolecta* PSI-LHCI supercomplex in Fe-starved conditions isolated by the GraFix strategy. (A) Flowchart showing all 3D classification and refinement steps used to determine the overall Fe-starved *D. tertiolecta* PSI-LHCI supercomplex using RELION (v.3.0).

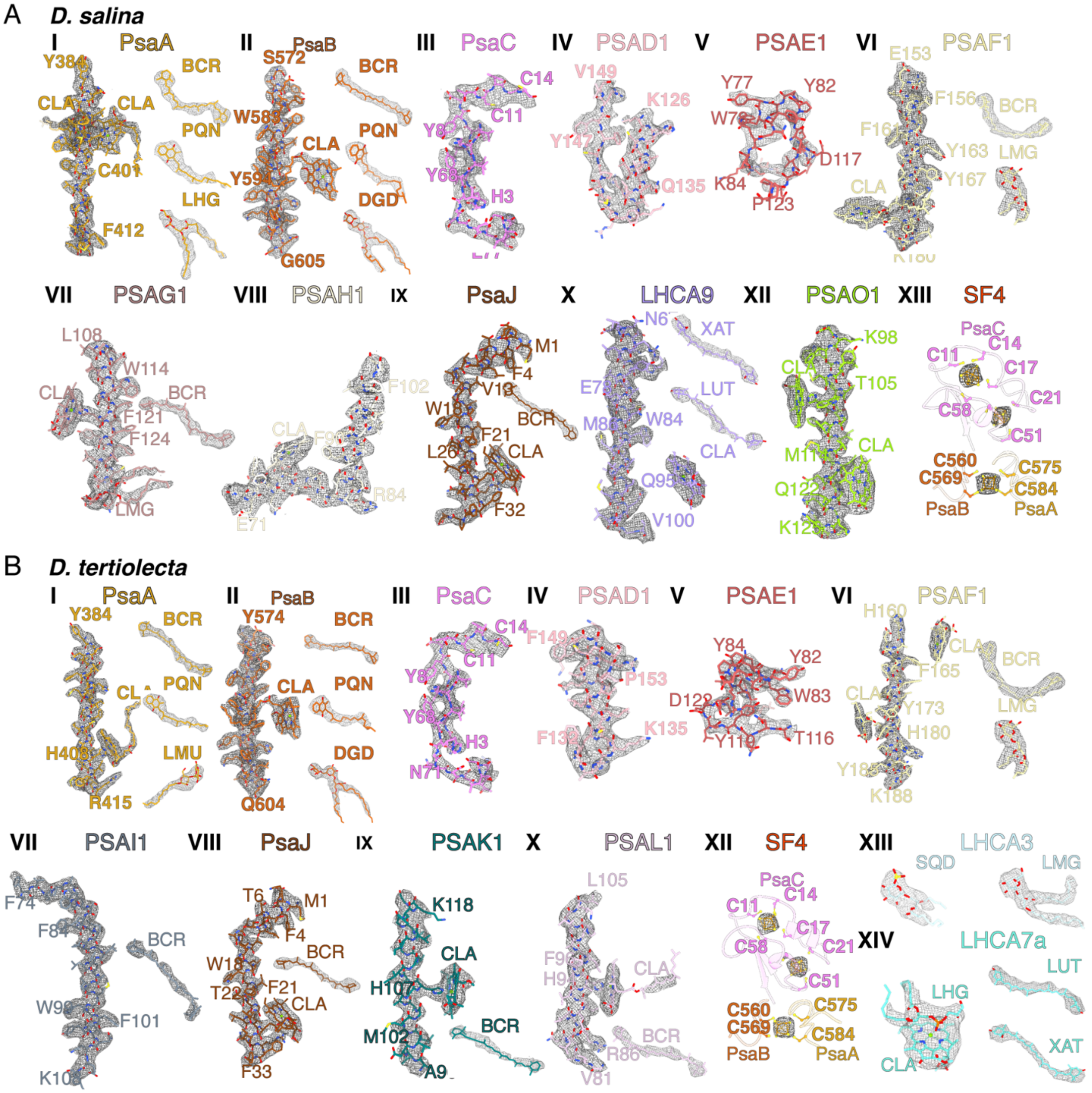

Fig. S13. Model fitting of the *Dunaliella* spp. PSI-LHCI$_2$ supercomplexes. (A and B) Model fitting of the PSI-LHCI$_2$ supercomplexes in the cryo-EM density map for different subunits of the structure in *D. salina* (A) and *D. tertiolecta* (B).

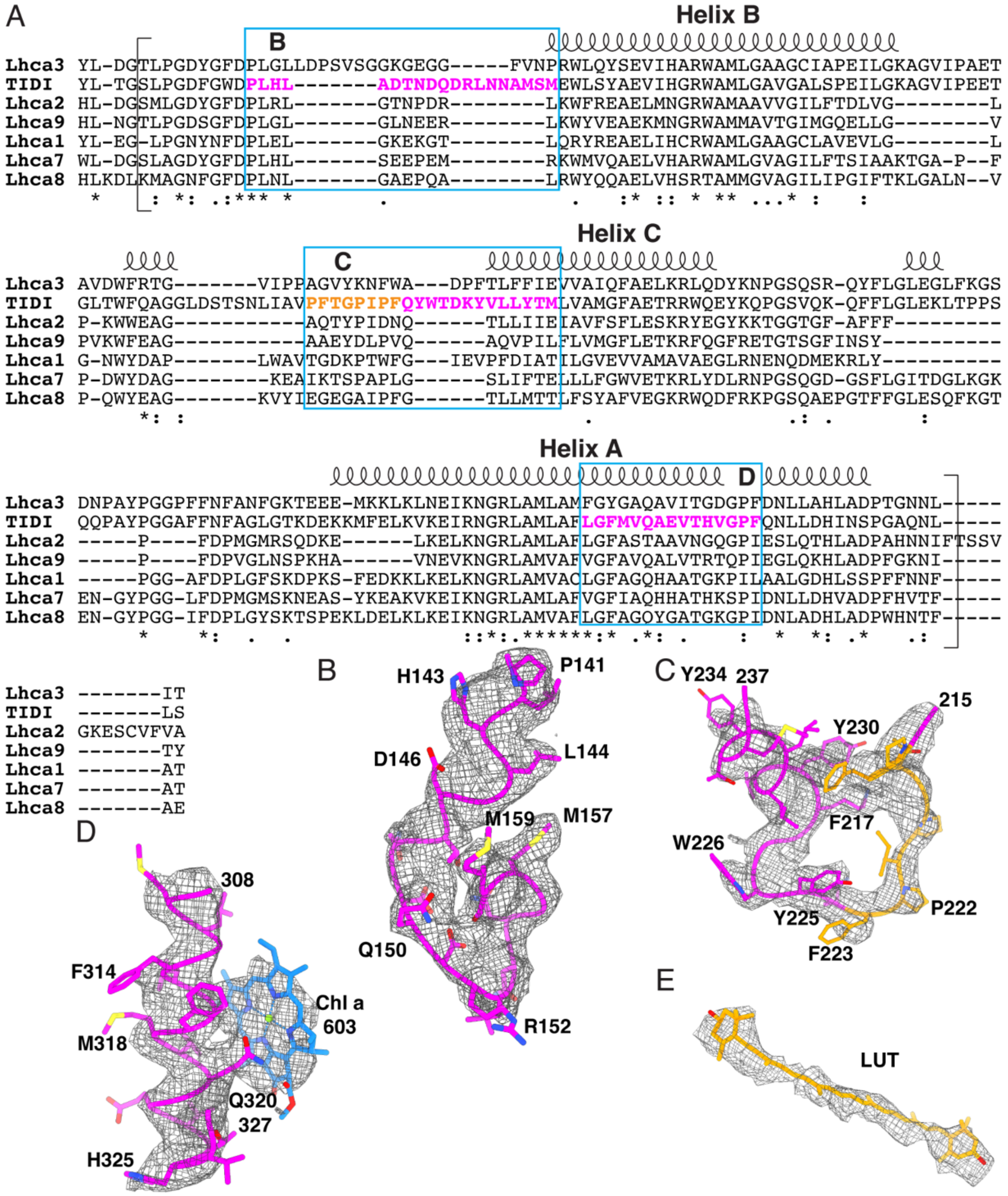

Fig. S14. Molecular details of *D. salina* TIDI1. (A) Multiple sequence alignment of *D. salina* LHCI subunits and TIDI1. The secondary structure is shown above the sequences. TIDI1 sequences best matching the cryo-EM density maps are shaded in magenta and boxed in blue frames. The amino acids in the conserved TIDI1 motif (PFXGX$_2$PF) are shaded in orange. (B–F) Cryo-EM density maps and structures of TIDI1 in regions best matching the TIDI1 sequences and pigment molecules.

A

```
                                                               Helix B
Lhca3 SDAAALKYL-DGTLPGDYGFDPLGLLDPTVSNGQGAGGFVNPRWLQYSEVIHARWAMLGAAGCIAPEILGKAG--VI
TIDI  SDERALSYL-TGSLPGDFGWDPLHLAD--TNDQDRLNNAMSMEWLSYAEVIHGRWAMLGAAGALSPEVLGKAG--VI
Lhca2 ATPPA--HL-DGSMLGDYGFDPLRLGT----------NPDRMKWFREAELTNGRWAMAAVVGILFTDLV------GL
Lhca9 VIPPP--HL-NGTLPGDSGFDPLGLGL----------NEERLKWYVEAEKTNGRWAMMAVTGILGQELL------GV
Lhca1 SEPKE--YL--NDLPGNFNFDPLELGK----------EKGTLQRYREAELIHCRWAMLGAAGCLAVEVL------GL
Lhca7 SQPPA--WL-DGSLAGDYGFDPLHLSE----------EPEMRKWMVQAELVHARWAMLGVAGILFTSIAAKNGA-PF
Lhca8 SQTPA--HLKDLKMAGNFGFDPLNLGA----------EPEALRWYQQAELVHSRTAMMAVAGILIPGLFTKLGALNV
               *      :  *:  .:*** *                  .    :*   :  *  **  ...*  :   :       .

                                                   Helix C
Lhca3 PAETAVDWFRTG-------VIPPAGVYKDFWA---DPFTLFFIEVVAIQFAELKRLQDYKNPGSQSR-QYFLGLEGL
TIDI  PKETGLTWFQAGGLDSSSNLIAVPFTGPIPFQYWTDQYTLLFTMLVAMGFAETRRWQEYKEPGSVQK-QFFLGLEKL
Lhca2 P-K----WWEAG-----------AQTYPIDNQ------TLAIIEIAVFAFLEAKRYEGYKKTGGTGF-AFF-----
Lhca9 PAK----WYEAG-----------AAEYDLPVQ------AQVPILFLVMGFLETKRFQGFRETGTSGFINSF------
Lhca1 G-N----WYDAP-------LWAVTGDKPTWFG---IEVPFDIATILGVEVVAMAVAEGLRNDNQDMEKRLY------
Lhca7 P-D----WYDAG--------KEAIKTSPAPLG------SLIFTELLLFGWVETKRLYDLRNPGSQGD-GSFLGITDG
Lhca8 P-Q----WYEAG-------KVYIEGEGAIPFG------TLLMSTLFSYAYFVEGKRWQDFRNPGSQAEPGTFFGLEGM
        .     *: :                             .   .                .: .     :

                                              Helix A
Lhca3 FKGSDNPAYPGGPFFNFANFGKTEAE-MKKLKLNEIKNGRLAMLAMFGYGAQAVITGDGPFDNLLAHLADPTGANLI
TIDI  TPPSQQPAYPGGGFFNFAGLGAKDEKQMFELKVKEIRNGRLAMLAFLGFMVQAEVTHVGPFQNLLDHINSPGAQNLL
Lhca2 ---------P----FDPMGMRSPEKE------LKELKNGRLAMLAFLGFASTAAVNGQGPIESLQTHLADPAHNNIF
Lhca9 ---------P----FDPVGLNSPKHA------VNEVKNGRLAMVAFIGFAMQALVSRTQPIEGLQKHLADPFGKNIT
Lhca1 ---------PGG-AFDPLGFSKDPKS-FEDKKLKELKNGRLAMVACLGFAGQHAATGKPILEALGDHLSSPFFNNFA
Lhca7 LKGKEN-GYPGG-LFDPMGMSKNEAS-FKEAKVKEIKNGRLAMLAFVGFIAQHHATHKSPIDNLVDHVADPFHVTFA
Lhca8 FKGTDN-GYPGG-IFDPLGYSKTSPEKLDELKLKEIKNGRLAMVAFLGFAGQYSATGKGPIDNLADHLADPWHNTFA
                  *     *:  .                ::*:.******:* .*:       .     :: *   *: .*   .:

Lhca3 TN-------
TIDI  SR-------
Lhca2 TSSVGKESC
Lhca9 YF-------
Lhca1 TN-------
Lhca7 TN-------
Lhca8 EN-------
```

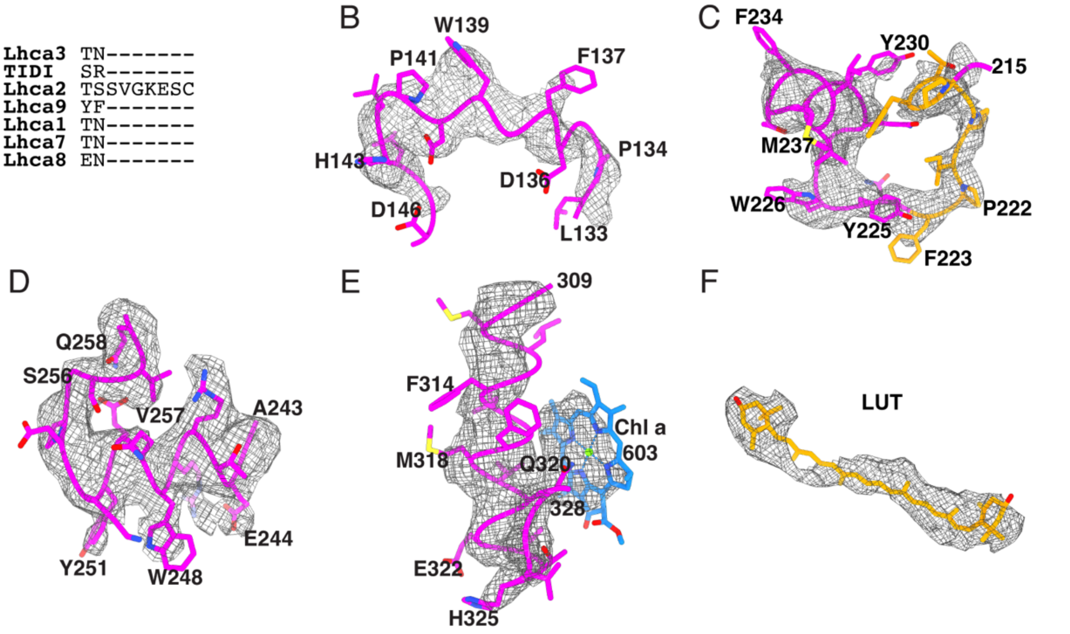

Fig. S15. Molecular details of *D. tertiolecta* TIDI1. (A) Multiple sequence alignment of *D. tertiolecta* LHCI subunits and TIDI1. The secondary structure is shown above the sequences. TIDI1 sequences best matching the cryo-EM density maps are shaded in magenta and boxed in blue frames. The conserved TIDI1 motif is shaded in orange. (B–F) Cryo-EM density maps and structures of TIDI in regions best matching the TIDI1 sequences and pigment molecules.

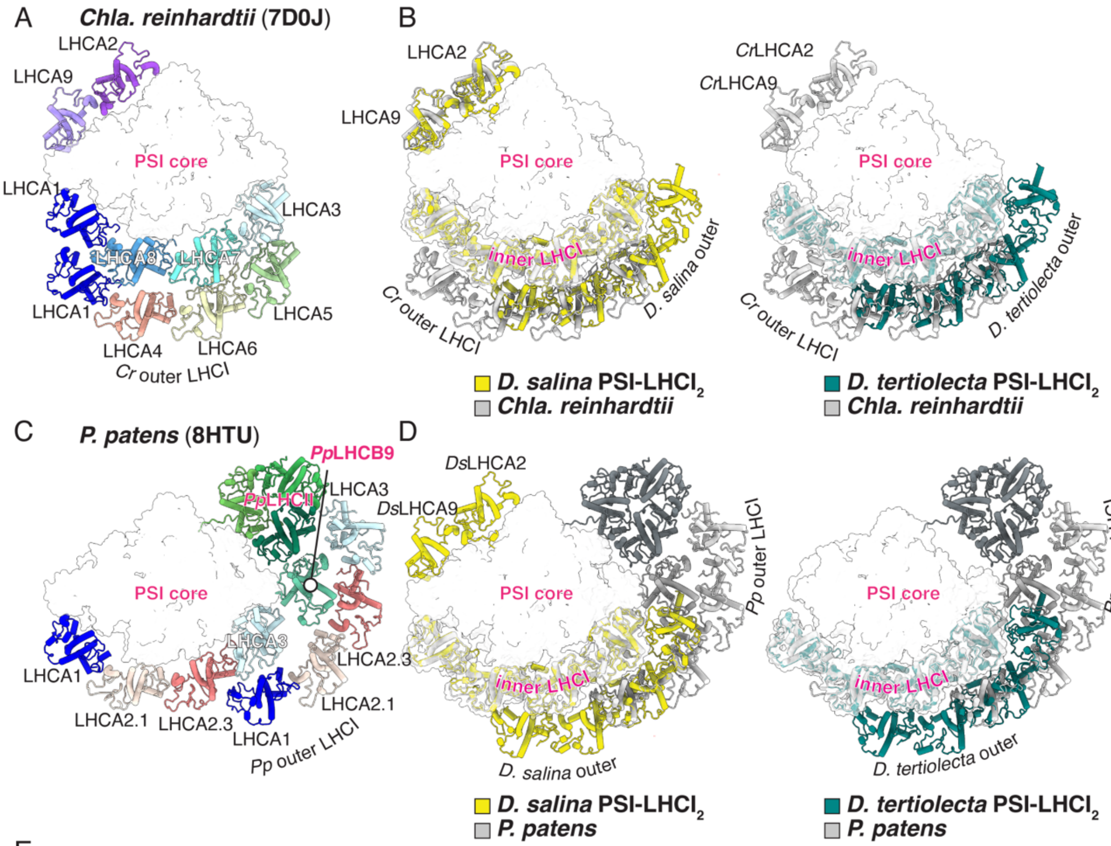

E

```
DsB_TIDI1  FLGFMVQAEV-THVGPFQNLLDHINSP------------------------------------------------
Dte_TIDI1  FLGFMVQAEV-THVGPFQNLLDHINSP------------------------------------------------
DsB_LHCA1  CLGFAGQHAA-TGKPILAALGDHLSSPFFNNFATNG-----------VSVPG-----------------------
Dte_LHCA1  CLGFAGQHAA-TGKPILEALGDHLSSPFFNNFATNG-----------VSVPGV----------------------
PpLHCA1    FLGFAVQAIAYPGTGPLENLKTHLADPWHNTIAH-------------VIIPRSVL--------------------
CrLHCA5    YMAFILQAQA-TGKGPLAALSAHLSNPFGNNILKNIGT---------CTVPHSVDVQGLTIPLTC----LWPGSQ
CrLHCA6    FVGFVMAAQV-TGKGPIAALQEHLADPWGTTIFSKAAVVPGQAVAPPCKIPASVSYKGIEIPTPCFLQGLWP---
DsB_LHCA7  FVGFIAQHHA-THKSPIDNLLDHVADPFHVTFATNG-----------VSIPHF----------------------
Dte_LHCA7  FVGFIAQHHA-THKSPIDNLVDHVADPFHVTFATNG-----------VSVPHFTEF-------------------
DsB_LHCA8  FLGFAGQYGA-TGKGPIDNLADHLADPWHNTFAENG-----------ISVPGLSAVEQAAANL------------
Dte_LHCA8  FLGFAGQYSA-TGKGPIDNLADHLADPWHNTFAENG-----------VSVPGLSAVEQAAAS-------------
            :.*      . .     :  *   *:  .*
```

Fig. S16. Comparison of LHCI tetramers from *Dunaliella* spp. compared to *Chla. reinhardtii* and *Phys. patens.* (A and C) Stromal view of *Cr*PSI-LHCI (PDB code 7D0J) (A) and large *Pp*PSI-LHCI (PDB code 8HTU) (C) supercomplex. (B and D) *Ds*PSI-LHCI$_2$ supercomplex (yellow, left) and *Dt*PSI-LHCI$_2$ supercomplex (teal, right) superimposed on *Cr*PSI-LHCI (gray) (B) or on *Pp*PSI-LHCI (gray) (D). The PSI core is shown either as cylinders (left) or surface view (middle, right). LHC subunits are shown as cylinders. (E) Multiple sequence alignment results of the C-terminal ends of the outer tetrameric LHCA proteins for *Dunaliella* spp., *P. patens* LHCA1, and *Chla. reinhardtii* LHCA5 and LHCA6.

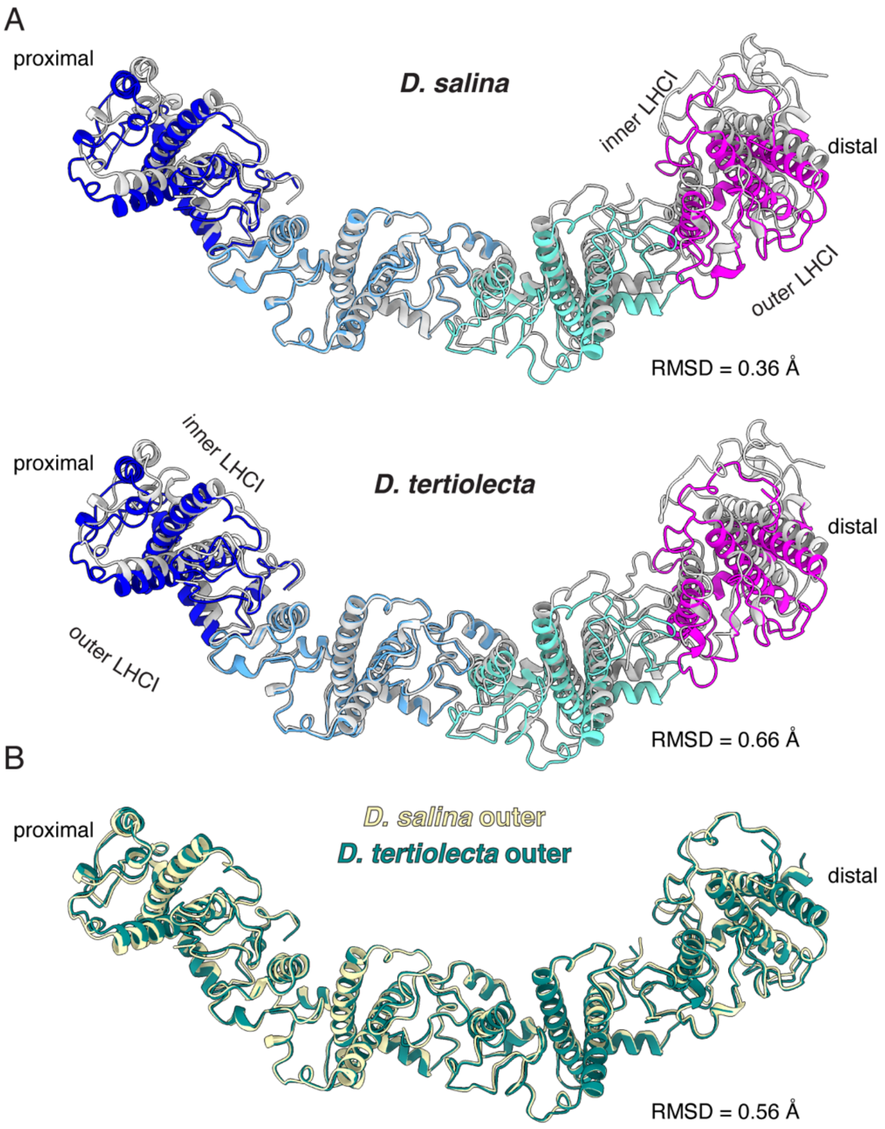

Fig. S17. Comparison of LHCI tetramers from *Dunaliella* spp. (A) Superimposition of inner LHCI tetramer (gray and LHCA3 as light blue) and outer LHCI tetramer (blue colored and TIDI1 as magenta) of the *Ds*PSI-LHCI$_2$ supercomplex (top) and *Dt*PSI-LHCI$_2$ supercomplex (bottom). (B) Superimposition of the outer LHCI tetramer of *Ds*PSI-LHCI$_2$ supercomplex (yellow) and *Dt*PSI-LHCI$_2$ supercomplex (teal).

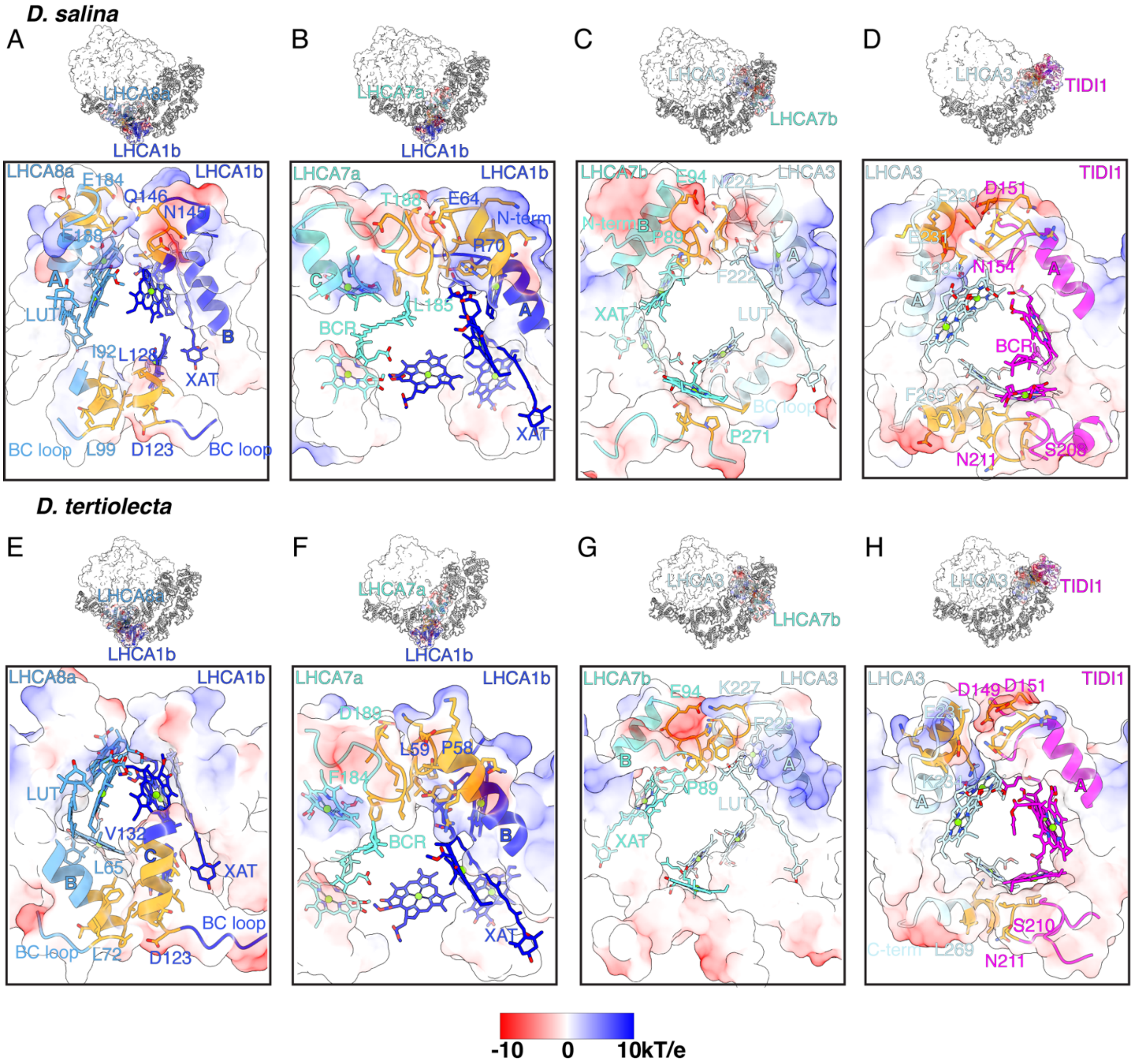

Fig. S18. Interactions between the LHCI tetramers of the *Dunaliella* spp. PSI-LHCI$_2$ supercomplexes. Electrostatic potential surface representation of the proposed interaction sites between the inner and outer LHC tetramers of the (A–D) *Dunaliella salina* and (E–H) *Dunaliella tertiolecta* PSI-LHCI$_2$ supercomplexes from Fe-starved conditions. Positively charged side chains are shown in blue and negatively charged residues are shown in red as indicated by the scale bar (–10 to 10 kT/e). The columbic potentials were computed using AMBER built-in Chimera X-1.7.1. Residues involved in the interaction between the outer and inner LHCI tetramer are highlighted in orange.

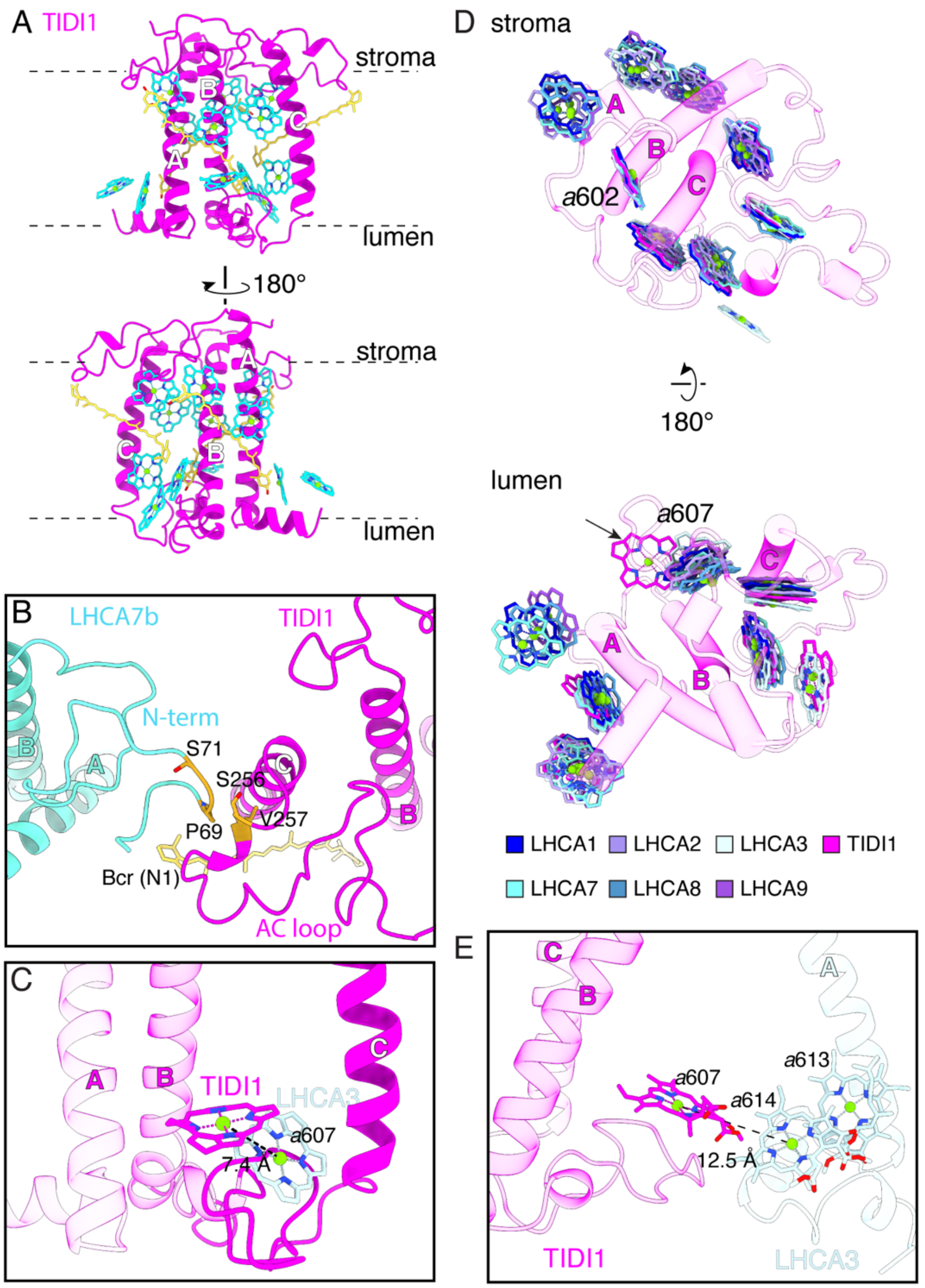

Fig. S19. The extended BC loop of *Dt*TIDI1 differently positions Chl *a*607. (A) Cartoon representation of *Dt*TIDI1. Chls are shown as cyan stick representations, with the central Mg atoms shown as spheres and numbered according to the conserved sites in spinach LHCII (PDB: 1RWT). Carotenoids are shown as yellow-colored stick representations. (B) Interactions between *Dt*TIDI1 and *Dt*LHCA7b in the outer LHCI tetramer. Residues involved in the interaction are highlighted in orange. (C) Comparison of *a*607$_{\mathrm{TIDI1}}$ (magenta) against position of *a*607$_{\mathrm{LHCA3}}$ (light blue) from *D. tertiolecta* on the lumenal side. Distance between molecules are shown as dashed lines. (D) Comparison of *Dt*TIDI1 Chl positions against Chl positions of *Dt*LHCA1, *Dt*LHCA2, *Dt*LHCA3, *Dt*LHCA7, *Dt*LHCA8, and *Dt*LHCA9 from *D. tertiolecta* on the stromal (top) and lumenal

side (bottom). (E) Side view of *D. tertiolecta* $a607_{TIDI1}$ (magenta) and its distance shown as to $a614_{LHCA3}$ (light blue).

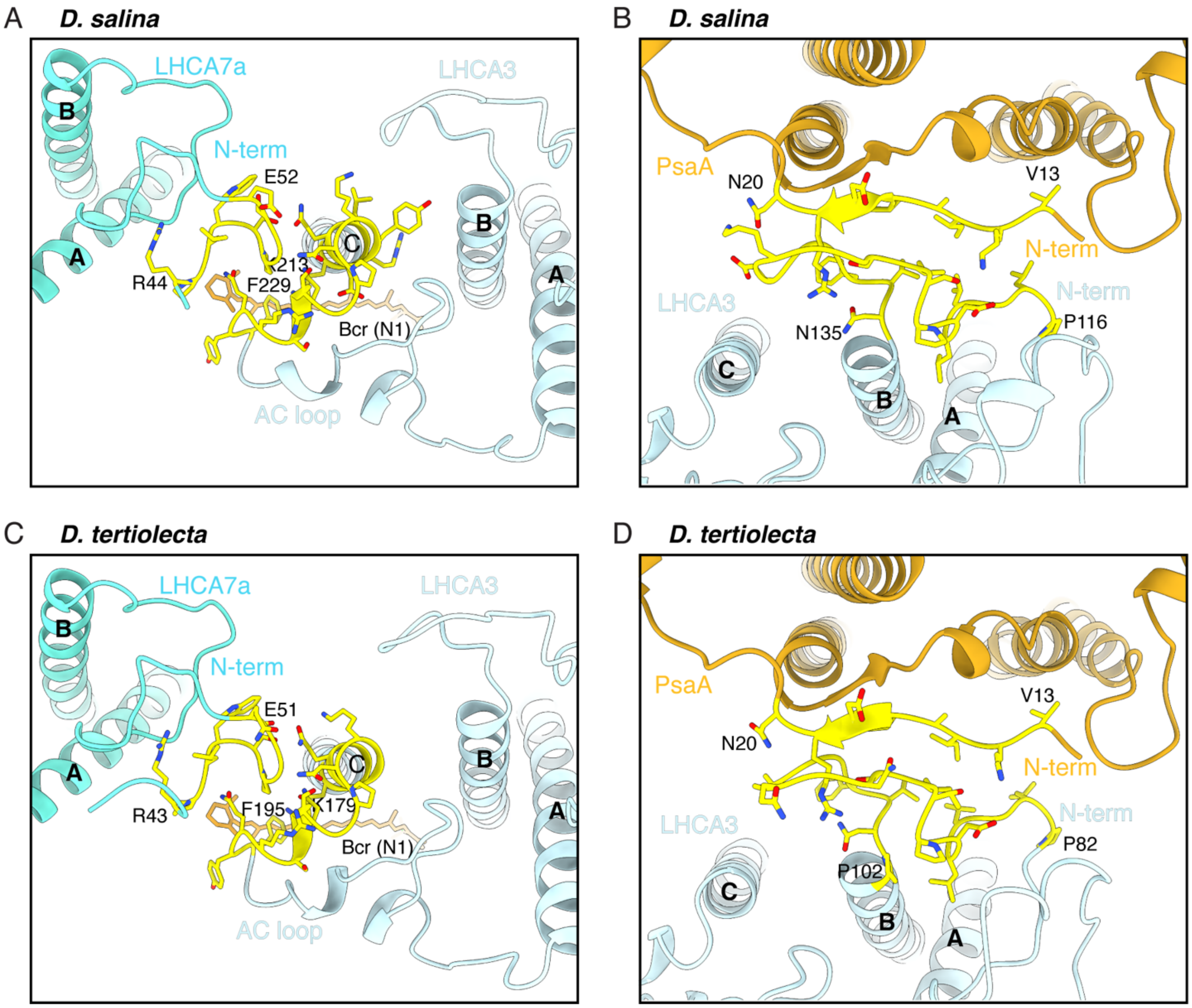

Fig. S20. Interactions between LHCA3 and either LHCA7a or PsaA. (A–D) Interactions between LHCA3 and LHCA7a in the inner LHCI tetramer (A and C) or between LHCA3 and PsaA in the PSI core (B and D) in either *Ds*PSI-LHCI$_2$ supercomplex or *Dt*PSI-LHCI$_2$ supercomplex. Residues involved in the interaction are shown as yellow stick representations.

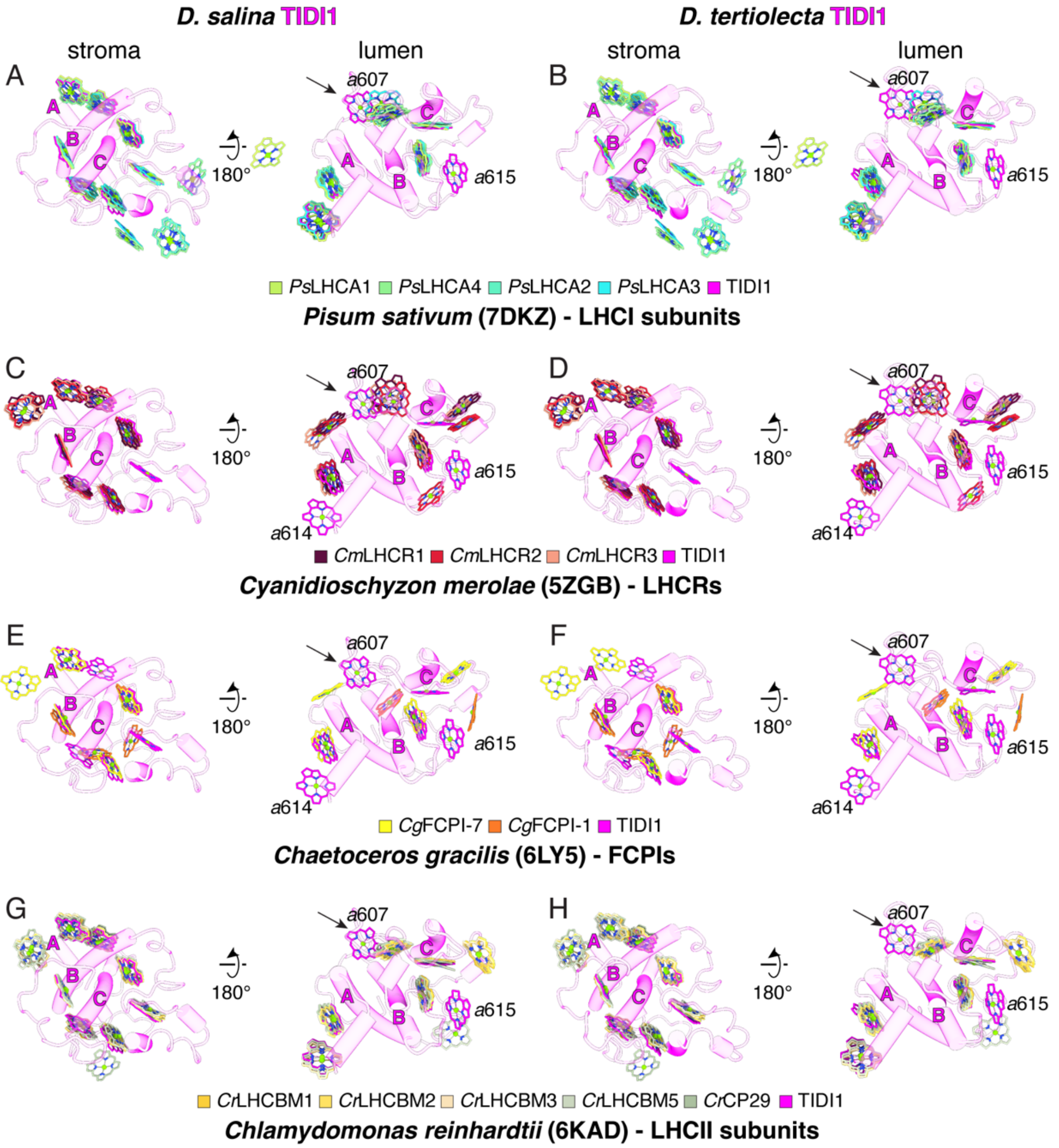

Fig. S21. Chlorophyll 607 is unique to *Dunaliella* TIDI1 across the green lineage. Comparison of chlorophyll arrangement in *Ds*TIDI1 is shown in magenta and higher plants (*P. sativum*, pea) (A and B), red algae (*Cyan. merolae*) (C and D), diatoms (*Chae. gracilis*) (E and F), and LHCII subunits (*Chla. reinhardtii*) (G and H) at the stromal layer (left) and at the lumenal layer (right) for *D. salina* and *D. tertiolecta*, respectively. Nomenclature of chlorophylls is based on LHCII structure. Transmembrane helices are labeled in all of the panels.

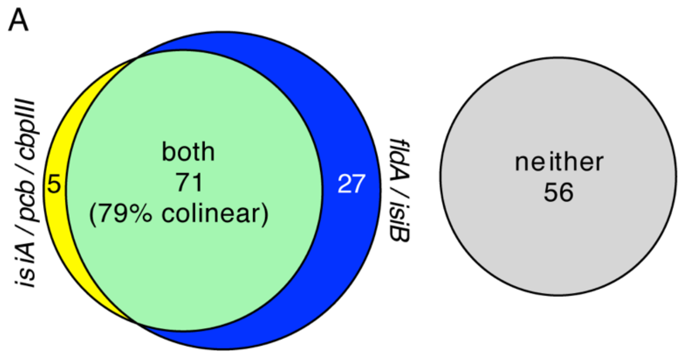

Fig. S22. The co-occurrence of *isiA* and *fldA* genes in cyanobacteria. (A)159 high-quality genome assemblies in the Cyanobacteriota lineage were surveyed for the presence of *isiA* (also called *cbp* and indistinguishable from *pcb*) and *fldA (*also called *isiB)* genes. Here, Euler plots show the percentage of species with one or more copies of *isiA*, with one or more copies of *fldA*, with both, or with neither. *isiA* and *fldA* genes were considered colinear if they were within 5 kb of each other on the same strand of the same contig in a species' genome. Supporting data are provided in Dataset S3.

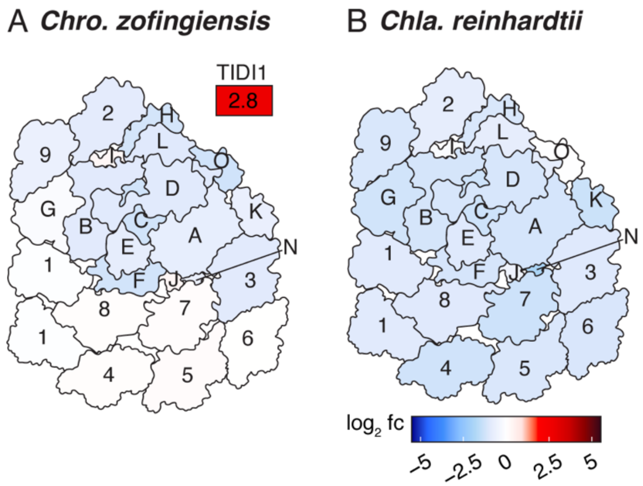

Fig. S23. Fe-starvation responses of PSI and LHCI subunits in diverse green algae. (A and B) The surface of PSI-LHCI supercomplexes colored by their $\log_2$ fold changes (fc) of the Fe-starved condition compared to the Fe-replete condition (red, increase; blue, decrease) for phototrophically grown *Chro. zofingiensis* (arrangement based on PDB:7D0J) (A) and *Chla. reinhardtii* (PDB:7D0J) (B).

Supporting Tables

Table S1

Data collection and processing	*D. salina* (+Fe) PSI-LHCI$_1$	*D. salina* (–Fe) PSI-LHCI$_2$	*D. tertiolecta* (+Fe) PSI-LHCI$_1$	*D. tertiolecta* (–Fe) PSI-LHCI$_2$	
	PDBid 9MH0 EMDB 48265	PDBid 9MGW EMDB 48262	PDBid 9MH1 EMDB 48266	PDBid 9MGZ EMDB 48264	
				N/A	GraFix
Microscope	UCB Titan Krios	UCB Titan Krios	UCB Titan Krios	UCB Titan Krios	UCB Titan Krios
Detector	K3	K3	K3	K3	K3
Magnification	81,000	81,000	81,000	81,000	81,000
Voltage (kV)	300	300	300	300	300
Electron Exposure (e$^-$/Å)	60	60	60	60	60
Defocus Range (µm)	–0.8 to –1.9	–0.8 to –1.9	–0.8 to –1.9	–0.8 to –1.9	–0.8 to –1.9
Pixal Size (Å)	1.05	1.05	1.05	1.05	1.05
Final symmetry imposed	C1	C1	C1	C1	C1
Initial particle images (#)	1,589,373	1,330,556	1,138,207	1,708,546	1,601,273
Final particle images (#)	98,784	126,839	120,321	123,355	217,073
Map resolution (Å)	2.9	2.9	2.1	2.7	2.8
FSC threshold	0.143	0.143	0.143	0.143	0.143
Map resolution range (Å)	1.5-5	1-4	1.5-5	1.5-5	1.5-5

Data collection and processing					
Initial model used (PDBid)	6SL5	6SL5	6SL5	6SL5	6SL5
Model resolution (Å)	2.9	2.9	2.1	2.7	2.8
FSC threshold	0.143	0.143	0.143	0.143	0.143
Model composition					
Non-hydrogen atoms					
Protein residues	3664	4642	3640	3918	
Ligand	291	362	270	284	
B factor (Å^2)					
Protein	91.69	126.89	109.18	131.02	
Ligand	96.19	131.72	109.70	137.57	
R.m.s deviations					
Bond lengths (Å) (# > 4σ)	0.01 (7)	0.01 (10)	0.01 (4)	0.01 (4)	
Bond angles (°) (# > 4σ)	1.26 (110)	1.31 (173)	1.23 (89)	1.22 (114)	
Validation					
MolProbity score	2.03	2.01	1.90	1.83	
Clashscore	14.39	13.43	12.50	11.65	
Poor rotamers (%)	0.58	0.59	0.83	0.87	
Ramachandran plot					
Favored (%)	94.76	94.56	95.81	96.34	
Allowed (%)	5.02	5.37	4.08	3.58	
Disallowed (%)	0.22	0.07	0.11	0.08	

Table S2. Cofactors in each subunit of the Fe-supplemented *Dunaliella* spp. PSI-LHCI$_1$ supercomplex.

Species	Subunit	Traced residues	Chls	Cars	Lipids	Others
D. salina	PsaA	740 (12-751)	43 *a*, 1 *a'*	7 βCR	3 LHG, 2 LMU, 1 OCD	1 PQ, SF4
	PsaB	732 (3-734)	41 *a*	7 βCR	1 DGD, 3 LHG, 1 SQD	1 PQ
	PsaC	80 (2-81)				2 SF4
	PSAD1	143 (60-202)				
	PSAE1	64 (60-123)				
	PSAF1	165 (68-232)	4 *a*	2 βCR	2 LHG, 3 PTY	
	PSAG1	105 (37-141)	3 *a*	1 βCR	2 LHG	
	**PSAH1**	65 (69-133)	2 *a*	1 βCR		
	PSAI1	41 (68-108)		2 βCR		
	PsaJ	41(1-41)	1 *a*	2 βCR	1 LHG	
	PSAK1	83 (41-123)	4 *a*	1 βCR		
	**PSAL1**	156 (47-203)	4 *a*	2 βCR	1 4RF, 1 DGD, 1 PTY	
	LHCA1a	197 (32-228)	12 *a*, 2 *b*	1 βCR, 1 XAT, 1 LUT	2 LHG, 2 LMG	
	LHCA7a	209 (63-271)	10 *a*, 4 *b*	1 βCR, 1 XAT, 1 LUT	1 DGD, 4 LHG, 2 LMG, 1 PTY	
	LHCA8a	226 (28-253)	11 *a*, 3 *b*	1 βCR, 1 XAT, 1 LUT	2 LHG, 1 LMK, 2 PTY	
	LHCA3	208 (112-319)	13 *a*, 1 *b*	3 βCR, 1 XAT, 1 LUT	1 DGD, 1 LHG, 2 LMG, 1 PTY, 2 SQD	
	LHCA2	222 (41-262)	11 *a*, 2 *b*	4 LUT	3 LHG, 2 PTY, 1 SQD, 1 3PH	
	LHCA9	187 (35-221)	11 *a*, 1 *b*	2 XAT, 1 LUT	1 LHG, 1 LMU, 1 PTY, 1 SQD	
D. tertiolecta	PsaA	740 (12-751)	43 *a*, 1 *a'*	7 βCR	3 LHG, 2 LMU, 1 PTY	1 PQ, SF4
	PsaB	734 (2-735)	41 *a*	7 βCR	1 DGD, 2 LHG, 1 LMU, 2 SQD	1 PQ
	PsaC	80 (2-81)				2 SF4
	PSAD1	143 (69-211)				
	PSAE1	68 (62-129)				
	PSAF1	165 (78-242)	4 *a*	2 βCR	1 LMG, 1 SQD, 1 PTY	
	PSAG1	105(36-140)	3 *a*	1 βCR	1 LMG	
	PSAH1	47 (70-116)	1 *a*			
	PSAI1	35 (15-49)		2 βCR		
	PsaJ	41(1-41)	1 *a*	2 βCR	1 LMU	
	PSAK1	83 (41-123)	4 *a*	1 βCR		
	PSAL1	138 (56-193)	2 *a*	1 βCR		
	LHCA1a	197 (32-228)	12 *a*, 2 *b*	1 βCR, 1 XAT, 1 LUT	1 LHG	
	LHCA7a	217 (39-255)	10 *a*, 4 *b*	1 βCR, 1 XAT, 1 LUT	3 LHG, 1 DGD, 1 SQD	
	LHCA8a	226 (28-253)	11 *a*, 3 *b*	1 βCR, 1 XAT, 1 LUT	3 LHG, 1 LMG, 1 LMU, 1 LMK, 1 PTY	
	LHCA3	226 (62-287)	13 *a*, 1 *b*	3 βCR, 1 XAT, 1 LUT	2 LMG, 2 SQD, 2 PTY	
	LHCA2	222 (39-260)	11 *a*, 2 *b*	2 LUT	2 LHG	
	LHCA9	187 (35-221)	11 *a*, 1 *b*	2 XAT, 1 LUT	2 LHG, 1 LMU, 1 PTY	

DGD, digalactosyldiacyl glycerol; LHG, dipalmitoyl phosphatidylglycerol; LMU, dodecyl-alpha-d-maltoside;PTY, phosphatidyl glycerol; SQD, sulfoquinovosyldiacyl glycerol; 3PH, phosphatidic acid; 4RF, tripalmitoylglycerol; OCD, octadecanal; PQN, phylloquinone; Chl, chlorophyll; Cars, carotenoids; SF4, sulfur-iron cluster; Red color denotes differences from the *Dt*PSI-LHCI$_1$ supercomplex

Extended Table 3. Cofactors in each subunit of the Fe-starved *D. salina* PSI-LHCI$_2$ supercomplex.

Species	Subunit	Traced residues	Chls	Cars	Lipids	Others
D. salina	PsaA	740 (12-751)	43 *a*, 1 *a'*	7 βCR	3 LHG, 2 LMU,	1 PQ, 1 SF4
	PsaB	732 (3-734)	41 *a*	6 βCR	1 DGD, 2 LHG, 2 PTY	1 PQ
	PsaC	80 (2-81)				2 SF4
	PSAD1	143 (60-202)				
	PSAE1	64 (60-123)				
	PSAF1	165 (68-232)	4 *a*	2 βCR	1 LMG, 1 PTY	
	**PSAG1**	105 (37-141)	3 *a*	1 βCR	1 LMG	
	**PSAH1**	98 (36-133)	2 *a*	1 βCR		
	PSAI1	41 (68-108)		2 βCR		
	PsaJ	41(1-41)	1 *a*	2 βCR		
	PSAK1	83 (41-123)	4 *a*	1 βCR		
	**PSAL1**	159 (44-203)	4 *a*	2 βCR	1 PTY, 1 4RF	
	**PSAO1**	88 (42-129)	3 *a*	1 βCR, 1 LUT		
	LHCA1a	197 (32-228)	12 *a*, 2 *b*	1 βCR, 1 XAT, 1 LUT	2 LHG, 1 LMG, 1 LMU, 1 3PH	
	LHCA7a	209 (43-251)	10 *a*, 4 *b*	1 βCR, 1 XAT, 1 LUT	1 DGD, 4 LHG, 1 LMG, 1 PTY	
	LHCA8a	226 (28-253)	11 *a*, 3 *b*	1 βCR, 1 XAT, 1 LUT	1 LHG, 1 LMK, 2 PTY	
	LHCA3	230 (90-319)	13 *a*, 1 *b*	3 βCR, 1 XAT, 1 LUT	2 DGD, 1 LHG, 2 LMG, 1 PTY, 1 SQD	
	LHCA1b	196 (32-227)	12 *a*, 2 *b*	1 βCR, 1 XAT, 1 LUT	2 LHG, 1 PTY	
	LHCA7b	209 (43-251)	10 *a*, 4 *b*	1 βCR, 1 XAT, 1 LUT	1 LHG, 3 PTY	
	LHCA8b	223 (29-251)	11 *a*, 3 *b*	1 βCR, 1 XAT, 1 LUT		
	**TIDI1**	205 (148-352)	12 *a*, 1 *b*	1 βCR, 1 XAT, 1 LUT	1 LHG	
	**LHCA2**	221 (41-261)	11 *a*, 2 *b*	4 LUT	2 LHG, 2 PTY, 1 SQD, 1 3PH	
	**LHCA9**	187 (35-221)	11 *a*, 1 *b*	2 XAT, 1 LUT	2 LHG, 1 LMU, 1 PTY	

DGD, digalactosyldiacyl glycerol; LHG, dipalmitoyl phosphatidylglycerol; LMU, dodecyl-alpha-d-maltoside;PTY, phosphatidyl glycerol; SQD, sulfoquinovosyldiacyl glycerol; 3PH, phophatidic acid; 4RF, tripalmitoylglycerol; PQN, phylloquinone; Chl, chlorophyll; Cars, carotenoids; SF4, sulfur-iron cluster; Red color denotes differences from the *Dt* PSI-LHCI$_2$ supercomplex; Red boxes denotes subunits absent in the *Dt*PSI-LHCI$_2$ supercomplex

Table S4. Cofactors in each subunit of the Fe-starved *D. tertiolecta* PSI-LHCI$_2$ supercomplex.

Species	Subunit	Traced residues	Chls	Cars	Lipids	Others
D. tertiolecta	PsaA	740 (12-751)	43 *a*, 1 *a'*	7 βCR	2 LHG, 2 LMU	1 PQ, SF4
	PsaB	734 (2-735)	41 *a*	6 βCR	1 DGD	1 PQ
	PsaC	80 (2-81)				2 SF4
	PsaD	143 (51-193)				
	PsaE	67 (43-109)				
	PsaF	165 (63-227)	4 *a*	2 βCR	1 LMG	
	PsaI	35 (15-49)		2 βCR		
	PsaJ	41(1-41)	1 *a*	2 βCR		
	PsaK	83 (41-123)	4 *a*	1 βCR		
	PsaL	138 (56-193)	2 *a*	1 βCR		
	LHCA1a	197 (32-228)	12 *a*, 2 *b*	1 βCR, 1 XAT, 1 LUT	3 LHG, 2 PTY	
	LHCA7a	217 (39-255)	10 *a*, 4 *b*	1 βCR, 1 XAT, 1 LUT	1 DGD, 2 LHG	
	LHCA8a	226 (28-253)	11 *a*, 3 *b*	1 βCR, 1 XAT, 1 LUT	1 LHG, 1 LMK, 2 PTY	
	LHCA3	226 (58-283)	13 *a*, 1 *b*	3 βCR, 1 XAT, 1 LUT	1 DGD, 2 LMG, 1 SQD	
	LHCA1b	197 (32-228)	12 *a*, 2 *b*	1 βCR, 1 XAT, 1 LUT	2 LHG	
	LHCA7b	209 (42-250)	10 *a*, 4 *b*	1 βCR, 1 XAT, 1 LUT	1 LHG	
	LHCA8b	224 (28-251)	11 *a*, 3 *b*	1 βCR, 1 XAT, 1 LUT	1 LHG	
	TIDI1	210 (130-339)	11 *a*, 1 *b*	1 βCR, 1 XAT, 1 LUT		

DGD, digalactosyldiacyl glycerol; LHG, dipalmitoyl phosphatidylglycerol; LMU, dodecyl-alpha-d-maltoside;PTY, phosphatidyl glycerol; SQD, sulfoquinovosyldiacyl glycerol; PQN, phylloquinone; Chl, chlorophyll; Cars, carotenoids; SF4, sulfur-iron cluster

Table S5. Sequence homology of *Dunaliella* spp. PSI-LHCI subunits

Subunit	Percent (%) identity
PsaA	100.0
PsaB	99.6
PsaC	100.0
PSAD1	92.7
PSAE1	90.0
PSAF1	92.5
PSAG1	87.2
PSAH1	90.3
PSAI1	88.0
PsaJ	100.0
PSAK1	86.8
PSAL1	90.9
PSAO1	91.4
LHCA1	93.9
LHCA2	91.6
LHCA3	95.8
LHCA7	94.5
LHCA8	93.7
LHCA9	94.6
TIDI1	89.1

Subunits that bind Chl are indicated in green text

Legends for supporting datasets

Dataset S1 (separate file): Proteomics values for *Dunaliella* spp. grown in either Fe-replete or Fe-starved medium. Spectral counts, protein abundance as $\log_2$ MASIC abundances of cells grown in either Fe-supplemented (15 µM Fe) or Fe-depleted (0.15 µM Fe) medium, and $\log_2$ fold change relative to the Fe-supplemented (15 µM Fe) condition.

Dataset S2 (separate file): Protein abundances of bioenergetic protein complexes. Spectral counts, protein abundance as $\log_2$ MASIC abundances of cells grown in either Fe-replete (15 µM Fe) or Fe-starved (0.15 µM Fe) medium, and $\log_2$ fold change relative to the Fe-replete (15 µM Fe) condition. Transcript abundance (FPKM) from Davidi et al., 2023, in either Fe-replete (1.5 µM Fe) or Fe-starved (0 µM Fe).

Dataset S3 (separate file): Co-occurrence of *isiA* and *fldA* in cyanobacteria. A systematic survey was conducted of reference genome assemblies in the NCBI genome database to identify the percentage of species that have both *isiA*/*pcb*/*cbp* genes and *fldA*/*isiB* genes. This data is provided in support of Figure S22. See Supplemental Methods for additional details.

SI References

1. C. Hui, *et al.*, Simple steps to enable reproducibility: culture conditions affecting *Chlamydomonas* growth and elemental composition. *The Plant Journal* **111**, 995–1014 (2022).

2. R. J. Porra, W. A. Thompson, P. E. Kriedemann, Determination of accurate extinction coefficients and simultaneous equations for assaying chlorophylls *a* and *b* extracted with four different solvents: verification of the concentration of chlorophyll standards by atomic absorption spectroscopy. *Biochimica et Biophysica Acta (BBA) - Bioenergetics* **975**, 384–394 (1989).

3. T. Varsano, S. G. Wolf, U. Pick, A chlorophyll *a*/*b*-binding protein homolog that is induced by iron deficiency is associated with enlarged photosystem I units in the eucaryotic alga *Dunaliella salina*. *Journal of Biological Chemistry* **281**, 10305–10315 (2006).

4. H. W. Liu, *et al.*, Chlamydomonas cells transition through distinct Fe nutrition stages within 48 h of transfer to Fe-free medium. *Photosynth Res* (2024). https://doi.org/10.1007/s11120-024-01103-8.

5. S. Merchant, B. R. Selman, Identification of the α and β subunits of the chloroplast coupling factor one in *Chlamydomonas reinhardi*. *European Journal of Biochemistry* **137**, 373–376 (1983).

6. A. M. Terauchi, *et al.*, Pattern of expression and substrate specificity of chloroplast ferredoxins from *Chlamydomonas reinhardtii*. *Journal of Biological Chemistry* **284**, 25867–25878 (2009).

7. L. Davidi, *et al.*, Pumping iron: A multi-omics analysis of two extremophilic algae reveals iron economy management. *Proceedings of the National Academy of Sciences* **120**, e2305495120 (2023).

8. J. Farah, F. Rappaport, Y. Choquet, P. Joliot, J. D. Rochaix, Isolation of a *psaF*-deficient mutant of *Chlamydomonas reinhardtii*: efficient interaction of plastocyanin with the photosystem I reaction center is mediated by the PsaF subunit. *The EMBO Journal* **14**, 4976–4984 (1995).

9. M. Broderson, K. K. Niyogi, M. Iwai, Macroscale structural changes of thylakoid architecture during high light acclimation in *Chlamydomonas reinhardtii*. *Photosynth Res* (2024). https://doi.org/10.1007/s11120-023-01067-1.

10. A. Jia, Y. Zheng, H. Chen, Q. Wang, Regulation and Functional Complexity of the Chlorophyll-Binding Protein IsiA. *Front. Microbiol.* **12** (2021).

11. M. Chen, T. S. Bibby, Photosynthetic Apparatus of Antenna-reaction Centres Supercomplexes in Oxyphotobacteria: Insight through Significance of Pcb/IsiA Proteins. *Photosynth Res* **86**, 165–173 (2005).

12. M. Chen, Y. Zhang, R. E. Blankenship, Nomenclature for membrane-bound light-harvesting complexes of cyanobacteria. *Photosynth Res* **95**, 147–154 (2008).
